# Development and validation of a multi-modal MRI-based deep learning framework for differentiation of intraspinal tumors (ISMF-Net)

**DOI:** 10.1016/j.eclinm.2025.103636

**Published:** 2025-11-13

**Authors:** Qianhui Zhang, Jianxin Yang, Qi Guo, Xueqin Chao, Yiyao Sun, Chunna Yang, Fangyuan Zhang, Bo Huang, Huanhuan Chen, Xiran Jiang

**Affiliations:** a Department of Biomedical Engineering, School of Intelligent Medicine, China Medical University, Liaoning, 110122, PR China; b Department of General Surgery, Shengjing Hospital, Shenyang, 110004, PR China; c Department of Pathology, Liaoning Cancer Hospital and Institute, Liaoning, 110042, PR China; d Department of Oncology, Shengjing Hospital, Shenyang, 110004, PR China; e Department of Clinical Epidemiology, Shengjing Hospital, Shenyang, 110004, PR China

**Keywords:** Intraspinal tumor, MRI, Deep learning, Cross-modal feature fusion

## Abstract

**Background:**

Intraspinal tumors (ISTs) pose diagnostic challenges due to their complex anatomy and reliance on subjective clinical imaging interpretation, while artificial intelligence-based methods offer significant potential for non-invasive ISTs diagnosis. We aimed to develop and validate an MRI-based deep learning model for IST differentiation.

**Methods:**

We conducted a retrospective study across three hospitals in China between January 2010 and July 2025. This retrospective study included 1004 patients diagnosed with ISTs, categorized into Schwannoma (SCN), Meningioma (MNG), Astrocytoma (AST), Ependymoma (EPN), and Metastasis (MET). Patients from Center 1 (n = 723) formed the internal dataset, which was randomly divided into training, validation and internal test sets at a 7:1:2 ratio. Additionally, 281 patients from Centers 2 and 3 were used as an external test set. All patients underwent preoperative spinal MRI, including sagittal T1-weighted (T1W), T2-weighted (T2W), T2 fat-suppressed (T2FS) and axial T2W sequences. We proposed the ISMF-Net that integrates features from sequences and clinical data to differentiate ISTs. Diagnostic improvements with model assistance were evaluated through an observer study involving radiologists of different levels of experience.

**Findings:**

Among 1004 patients, 22.6% had SCN, 21.8% had MNG, 14.9% had AST, 18.2% had EPN, and 22.5% had MET. The proposed ISMF-Net achieved micro-ACC of 0.859 [0.827, 0.889], 0.849 [0.826, 0.874], and 0.821 [0.801, 0.841] on the validation, internal test, and external test sets, respectively, outperforming existing methods. The highest performance was observed for SCN (F1: 0.905 [0.879, 0.930]–0.940 [0.910, 0.968]), while MET showed lower performance (F1: 0.757 [0.724, 0.790]–0.804 [0.746, 0.858]). Model assistance significantly improved diagnostic performance across all radiologists, with junior radiologists benefiting the most, showing increases of 7.9%, 20.1% and 4.9% in accuracy, sensitivity, and specificity, respectively. Additionally, preference analysis confirmed strong alignment between the model's predictions and radiologists' diagnostic tendencies.

**Interpretation:**

This study proposes a multi-modal DL approach integrating MRI and clinical data to improve IST diagnosis, providing a valuable tool for enhancing diagnostic accuracy in clinical practice.

**Funding:**

This study was funded by the General Program from Department of Education of Liaoning Province (JYTMS20230132), the National Natural Science Foundation of China (82304235) and the 10.13039/501100002858China Postdoctoral Science Foundation (2024M753641).

**Research in context**

**Evidence before this study**

We searched PubMed from the inception of the database to September 13, 2025, for research articles using the search terms “AI” or “artificial intelligence” or “deep learning” or “machine learning” and “intraspinal tumor” or “spinal cord tumor” or “intramedullary tumor” or “extramedullary tumor” or “ISTs” and “MRI” or “magnetic resonance imaging,” without language restrictions. A total of seven studies were identified that developed and validated artificial intelligence (AI)-assisted MRI diagnostic models for intraspinal tumors (ISTs). However, most focused on binary classification between limited tumor types and used single-plane or single-sequence magnetic resonance imaging (MRI) data. Few studies explored multimodal integration or modeling of the anatomical complexity of the spinal canal. Furthermore, current methods often rely on simplistic feature fusion strategies and fail to fully exploit the potential of multimodal MRI and anatomical context.

**Added value of this study**

Based on a comprehensive literature review, we developed ISMF-Net, a novel end-to-end deep learning (DL) framework that integrates sagittal, axial MRI, and clinical data, representing the first attempt to achieve five-class differentiation of ISTs. To address the current limitations of AI research in this field, the model incorporates a multimode fusion mechanism, allowing it to effectively capture tumor-specific morphology and spatial dependencies. Trained and validated on multi-center datasets, ISMF-Net demonstrated robust and consistent diagnostic performance, surpassing previous models in accuracy and generalizability. This study represents the first large-scale, multi-center development of an AI system specifically designed for comprehensive MRI-based IST classification.

**Implications of all the available evidence**

Our study demonstrates that ISMF-Net provides a reliable and efficient tool for automated ISTs differentiation across diverse anatomical locations and imaging conditions. The model can be deployed in hospitals with high workloads or limited expertise, supporting accurate and standardized diagnosis. By leveraging automated multimodal integration, ISMF-Net has the potential to streamline diagnostic workflows, improve inter-observer consistency, and deliver expert-level diagnostic performance in clinical practice.

## Introduction

Intraspinal tumors (ISTs) represent a significant challenge in clinical practice due to their potential to cause severe neurological damage and paralysis.^1^ The complex presentation and high misdiagnosis risk of ISTs impose substantial burdens on patients and healthcare systems, highlighting the need for early and accurate diagnosis to improve treatment outcomes and prognoses.^2^ However, current methods rely heavily on histopathological biopsy, which is invasive, complex, and carries inherent risks.^3^

Magnetic Resonance Imaging (MRI), renowned for its superior soft tissue resolution and inherent multi-modal capabilities, has become an indispensable non-invasive diagnostic tool in the evaluation of ISTs.^4^ Each individual sequence offers distinct diagnostic insights: T1-weighted (T1W) effectively delineates anatomical structures and lesion boundaries, T2-weighted (T2W) demonstrates high sensitivity to edema and intramedullary changes and T2-fat suppression (T2-FS) significantly enhances lesion contrast by suppressing fat signals, thereby optimizing contrast between the lesion and surrounding tissues. While individual MRI sequences provide valuable insights, they often fail to fully capture the multi-dimensional complexity of lesions, particularly in cases with ambiguous boundaries or overlapping imaging features, posing significant diagnostic challenges.^5^ The complementary information across multiple sequences offers a solid foundation to overcoming these limitations, enabling more precise lesion identification and characterization.^6^ Developing an advanced multi-modal fusion framework is therefore critical to improve diagnostic accuracy and holds significant clinical value.

Deep learning (DL) has achieved significant advancements in medical image analysis,^7^ and has been applied to the study of various intraspinal diseases, particularly in the field of ISTs. Convolutional neural network (CNN) models have demonstrated promising performances in differentiating specific pairs of ISTs, such as schwannoma (SCN) vs. meningioma (MNG),^8^, ^9^, ^10^ schwannoma (SCN) vs. ependymoma (EPN),^11^ and astrocytoma (AST) vs. ependymoma (EPN).^12^ However, the existed researches only focused on binary tasks limited to selected tumor pairs, which fails to reflect the broader pathological diversity encountered in routine clinical settings, thereby limiting their clinical applicability.

In addition, prior studies predominantly employed CNN architectures without structural adaptation to the morphological complexity of ISTs, resulting in increased computational burden and limited diagnostic performance. In particular, existing approaches suffer from methodological limitations in extracting and integrating multi-modal features. Most fail to effectively incorporate both multi-sequence MRI and relevant clinical data, thereby limiting the exploitation of complementary information across modalities. Moreover, current fusion frameworks insufficiently capture inter-modality dependencies and provide limited interpretability, which constrains clinical trust and application potential. These challenges highlight the need for further optimization in deep feature fusion, multi-modal information integration. To address the limitations, this study proposes ISMF-Net, which introduced the Dynamic Snake Convolution (DSConv)^13^ to adapt to the complex anatomical morphological characteristics of the spinal canal and improve the model's ability to capture subtle anatomical variations and fine structural features. ISMF-Net integrates multi-sequence, multi-plane MRI data and clinical information, aiming to utilize complementary information across different modalities to improve diagnostic performance of major types of ISTs. Specifically, the key contributions of this study include:

1 Proposed a Global-Local Attention Fusion Module (GLAFM) that integrates global contextual information from Transformer encoders with local structural details extracted by CNNs, improving feature representation for IST differentiation.
2 Developed Sequence- (SFM) and Multi-plane Fusion (MIM) modules to integrate complementary information across multi-sequence and multi-plane MRI data (T1W, T2W, T2-FS; sagittal and axial), thereby enriching modality-level representation and facilitating more comprehensive characterization of ISTs.
3 Conducted an observer study involving a radiologist group to evaluate the impact of ISMF-Net assistance on diagnostic performance, aiming to explore its potential for clinical translation and decision support.

## Methods

### Datasets

**Fig. 1:**
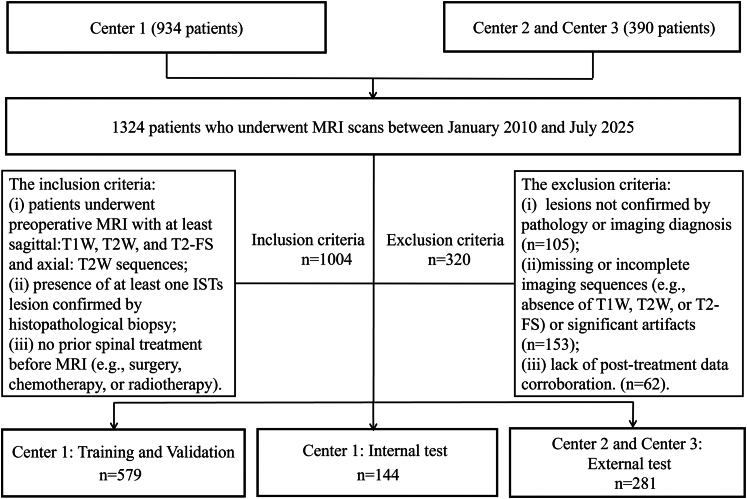
Patient inclusion and exclusion criteria. Abbreviations: ISTs, Intraspinal tumors; T1, T1-weighted imaging; T2, T2-weighted imaging; T2-fs, T2 fat-suppressed imaging.

We conducted a retrospective study across three hospitals in China between January 2010 and July 2025. The study was approved (approval number: 2025131) and informed consent was waived. Inclusion criteria were as follows: (1) patients underwent preoperative MRI with at least sag: T1W, T2W, and T2-FS and Axial: T2W sequences; (2) presence of at least one ISTs lesion confirmed by histopathological biopsy; and (3) no prior spinal treatment before MRI (e.g., surgery, chemotherapy, or radiotherapy). Exclusion criteria were as follows: (1) lesions not confirmed by pathology or imaging diagnosis; (2) missing or incomplete imaging sequences (e.g., absence of T1W, T2W, or T2-FS) or significant artifacts; and (3) lack of post-treatment data corroboration. A total of 1004 patients were included in the study. The primary dataset consisted of 723 cases from Center 1, which was randomly divided into a training set, a validation set and an internal test set at a 7:1:2 ratio. 281 cases from Center 2 and Center 3 served as the external test set. Fig. 1 illustrates the patient inclusion and exclusion process, as well as the data partitioning strategy. A detailed distribution across centers is provided in Supplementary Table S1.

### MRI acquisition and ground truth labels preparation

All 2D spinal MRI data were acquired using standard clinical protocols on 1.5T or 3.0T scanners. At Center 1, imaging was performed on a 3.0T GE scanner. Sagittal T1W, T2W, and T2-FS sequences were acquired with FA/TR/TE values of 80°/500/8 ms, 90°/2500/90 ms, and 90°/2800/97 ms, respectively; slice thickness (ST) was 3.0, 3.0, and 4.0 mm; field of view (FOV) was 320, 320, and 340 mm; matrix size was 512 × 512 for all sequences. Axial T2W was acquired with TR/TE = 3250/85 ms, matrix size = 512 × 512, slice thickness = 3.0 mm. At Center 2, imaging was conducted on a 1.5T Philips scanner. Sagittal T1W, T2W, and T2-FS sequences used FA/TR/TE of 90°/440/9 ms, 90°/2800/97 ms, and 90°/3000/130 ms, respectively; ST was 4.0, 3.0, and 3.0 mm; FOV 340, 340, and 240 mm; matrix 512 × 512. Axial T2W was acquired with TR/TE = 3315/108.72 ms, matrix size = 512 × 512, slice thickness = 4.0 mm. At Center 3, a 3.0T Siemens scanner was used. Sagittal T1W, T2W, and T2-FS sequences were acquired with FA/TR/TE of 90°/690/8 ms, 90°/2024/120 ms, and 90°/2000/120 ms, respectively; ST was 4.0 mm for all; FOV 300 mm; and matrix 200 × 273, 224 × 292, and 180 × 264, respectively. Axial T2W was acquired with TR/TE = 3945/76.3 ms, matrix size = 512 × 512, slice thickness = 4.0 mm.

### MRI image preprocessing

We first applied window width and window level normalization to standardize image intensities across three centers. The normalized images were then input into the YOLOv9^14^ network for automated lesion detection and localization. Based on the detection results, image cropping was performed to extract regions of interest. Two radiologists with 11 and 13 years of experience respectively, reviewed all detected lesions against the pathological findings. For incorrectly identified lesions, the radiologists manually re-cropped the original images using Microsoft Paint (Microsoft Corporation, Redmond, WA), ensuring the minimal region containing the tumor and the full extent of the spinal canal were preserved in both sagittal and axial views. All annotations were reviewed by a senior radiologist (20 years of experience) to ensure consistency and accuracy. The cropped images were resized to 224 × 224 pixels using bilinear interpolation to ensure consistent input dimensions. To address class imbalance, enhance model robustness and improve generalization across centers, augmentation methods such as random rotation, translation, and scaling were applied. Detailed YOLOv9 architecture and data augmentation process are provided in Supplementary Materials.

### Methodology

**Fig. 2:**
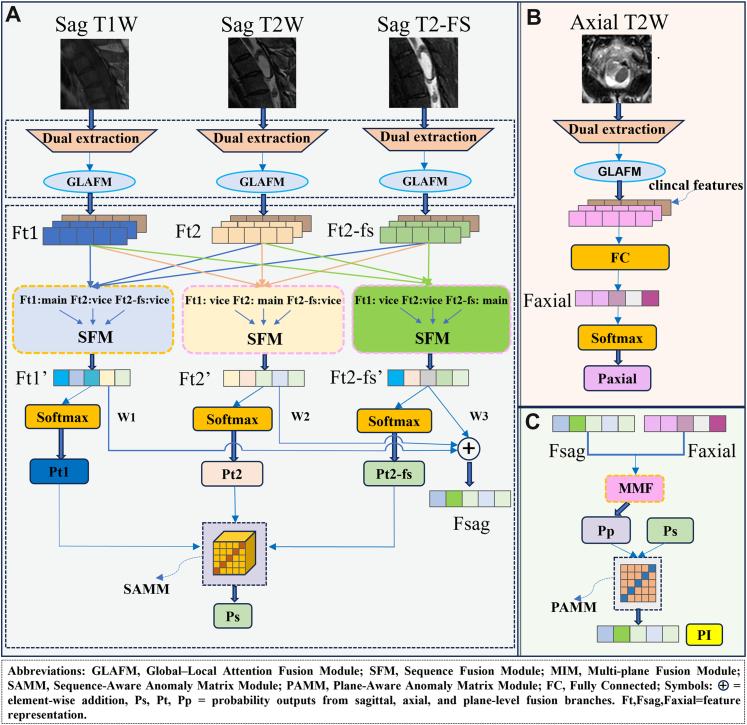
Overview of the proposed ISMF-Net, which integrates (A) Sequence-level fusion stage, (B) Axial feature extraction stage, and (C) Plane-level fusion stage.

In order to exploit the complementary information from multi-modal MRI sequences, multi-plane IST scans (sagittal and axial) and clinical features for accurate differentiation of different types of ISTs, we propose ISMF-Net (Fig. 2). ISMF-Net consists of three stages: a sequence-level fusion stage (Fig. 2A), a axial feature extraction stage (Fig. 2B) and plane-level fusion stage (Fig. 2C), which together enable comprehensive integration of multi-modal information. The input to ISMF-Net consists of three sagittal sequences (T1W, T2W, and T2-FS) and one axial T2W sequence.

(A) **Sequence-level fusion stage**

Three sagittal MRI sequences (T1, T2, and T2FS) are independently processed through a dual-branch feature extractor comprising a CNN and a transformer-base^15^ branch (Supplementary Figure S2). The CNN branch is implemented as an Adaptive ResNet,^16^ in which conventional convolution are replaced with DSConv^13^ and deformable convolution network (DCN)^17^ to better accommodate the elongated and complex anatomical structure of the spinal canal, thereby capturing local features, emphasizing texture and boundary details, while the transformer branch extracts global features, modeling long-range dependencies and contextual information. This design is intended to enable direction-aware feature extraction, enhance boundary delineation, and strengthen the representation of boundary-related features, while simultaneously capturing fine-grained local details (such as textures and boundaries) and global contextual information, thereby yielding more discriminative feature representations. The extracted local and global features are subsequently integrated through a Global-Local Attention Fusion Module (GLAFM) (Supplementary Figure S3), which employs parallel channel- and spatial-attention mechanisms to adaptively model both global contextual information and local boundary details, thereby generating more discriminative high-level representation (Ft1, Ft2, Ft2-fs) for each sequence. Subsequently, key clinical information (sex, age, and lesion location) was encoded and fused with Ft1, Ft2, and Ft2-FS to generate Ft1′, Ft2′, and Ft2-FS′, respectively.

To further integrate complementary information across different sequence while preserving sequence-specific characteristics, we propose a two-stage sequence fusion strategy. At the intermediate feature level, the Ft1′, Ft2′, and Ft2-fs′ was then feed to the Sequence Fusion Module (SFM) (Supplementary Figure S4), which employs the cross-attention mechanism to capture inter-sequence dependencies and produce fused representations with enhanced semantic expressiveness, generating enhanced features (Ft1′, Ft2′, Ft2-fs′). At the late probability-level fusion stage, to explore the relationships between ISTs and different sequences, each of these features undergoes two operations. First, these features are processed with a softmax to obtain probability scores (Pt1, Pt2, Pt2-fs), which are assembled into a Sequence-Aware Anomaly Matrix Module (SAMM) (Supplementary Figure S6), forming a 5 × 5 × 5 matrix that, through an attention-like weighting mechanism, to reflect the degree of abnormality across different modalities, yielding the sagittal level probability output Ps. The other operation applies a 1 × 1 convolution to Ft1′, Ft2′, and Ft2-fs’ to assign adaptive weights, producing the further fused feature Fsag, which is subsequently integrated with the axial feature Faxial to enable the subsequent two-stage plane-level fusion.

(B) **Axial feature extraction stage**:

The axial T2-weighted image is processed through the dual-branch feature extractor and the GLAFM module, followed by the embedding of key clinical information (sex, age, and lesion location) and a fully connected layer, to generate a discriminative representation Faxial and its corresponding prediction probability Paxial, thereby enriching cross-view information.

(C) **Plane-level fusion stage**:

To fully exploit the complementary anatomical information across different imaging planes, we also adopt a two-stage strategy in the plane-level fusion. At the feature level, we introduce the Multi-plane Fusion Module (MIM) (Supplementary Figure S5), which integrates the fused features from sagittal sequences (Fsag) with those from the axial plane (Faxial). By employing channel attention to reweight spatial information, the module preserves informative features while suppressing redundancy, enabling cross-plane feature alignment and producing the probability output Pp.

At the late probability-level fusion stage, Pp and Psag are used to construct a 5 × 5 Plane-Aware Anomaly Matrix Module (PAMM) (Supplementary Figure S6), for further probability-level fusion, yielding the final output probability PI. This process enhances the model's capacity to capture the spatial organization of spinal anatomy. Detailed module descriptions, architecture diagrams, and computational formulas are provided in the Supplementary Materials.

The loss function consists of three components: (1) Branch-level BCE loss ensures each branch learns effectively, which includes sag T1W, sag T2W, sag T2-FS and axial T2W; (2) Final output BCE loss optimizes the overall prediction; and (3) Focal Loss focuses on difficult types, improving performance on imbalanced datasets. This combination enhances the model's ability to handle class imbalance $y^{i}$ denotes the ground truth label, ${\hat{y}}_{\text{branch}}^{i}$ represents the prediction from each individual branch,${\hat{y}}^{i}$ represents the final integrated output of the model, and α is a weighting factor balancing branch-level and final-level losses. In our experiments, the value of α was set to 0.5. The focal loss parameter γ was set to 2.

$$L_{total}=\alpha \sum_{branch}\sum_{i=1}^{5}BCE\left(y_{,}^{i}{\hat{y}}_{branch}^{i}\right)\;+\sum_{i=1}^{5}BCE\left(y^{i},{\hat{y}}^{i}\right)+\sum_{i=1}^{5}FL\left(y^{i},{\hat{y}}^{i}\right)$$

### Performance metrics

We adopted a comprehensive set of evaluation metrics, including precision, recall, F1-score, micro-accuracy (Detailed calculation methods provided in Supplementary Materials), area under the ROC curve (AUC), accuracy, sensitivity, and specificity. These metrics were used to assess the diagnostic performance of both the ISMF-Net and the radiologists.

### Experimental

All experiments were conducted using Python 3.10.0 and PyTorch 2.0.1 with CUDA 11.8, on a workstation equipped with an Intel Core i9-12900 processor, 64 GB of RAM, and an NVIDIA GeForce RTX 3090 GPU with 24 GB of VRAM. The ISMF-Net was trained with optimized hyperparameters, including a batch size of 32 and an initial learning rate of 3e-4. A 5-epoch linear warm-up followed by a cosine annealing learning rate scheduler was applied to adjust the learning rate dynamically during training. The AdamW optimizer was adopted, with a weight decay coefficient set to 1e-4 to prevent overfitting, and the momentum decay rates were set to 0.90 and 0.99. The maximum number of training epochs was 500, with a minimum learning rate of 1e-6. Detailed parameter settings and descriptions of the strategies used to prevent overfitting are provided in Supplementary Materials.

### Observer study

An observer study was conducted to evaluate the clinical applicability of ISMF-Net. The radiologist group included two senior radiologists (20 and 30 years of working experiences), two intermediate radiologists (11 and 12 years), and three junior radiologists (1, 4 and 6 years). All independently reviewed the test dataset (n = 281; Center 2 and 3) to assess diagnostic performance and interpretability, with and without model assistance.

Radiologists independently diagnosed lesions and estimated probabilities without model assistance. After a 28 day interval, they re-evaluated the same cases with model support, using randomized image order in both sessions. Diagnostic performance (accuracy, sensitivity, specificity) before and after model assistance was compared across experience levels. Radiologists scored sequence- and plane-level contributions for each ISTs, which were correlated with model predictions. Model interpretability was assessed through Class Activation Mapping (CAM),^18^ generating lesion heatmaps to evaluate alignment between model attention and disease-relevant features. Agreement between the model and radiologists, as well as inter-rater consistency, was quantified using Cohen's kappa to validate clinical applicability.

### Statistics analysis

**Fig. 3:**
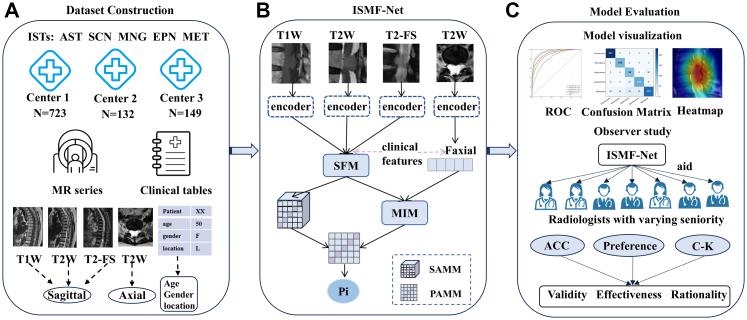
Overall framework of the study design. A: Dataset Construction, B: ISMF-Net, C: Model Evaluation. Abbreviations: ISTs, intraspinal tumors; T1, T1-weighted imaging; T2, T2-weighted imaging; T2-fs, T2 fat-suppressed imaging; SFM, sequence fusion module; MIM, multi-plane fusion module; SAMM, sequence-aware anomaly matrix module; PAMM, plane-aware anomaly matrix module.

Differences in baseline clinical characteristics among the training, internal test, and external test cohorts were assessed using one-way analysis of variance (ANOVA) for continuous variables and the chi-square test for categorical variables. Model performance across datasets was compared using the Wilcoxon rank-sum test, and 95% confidence intervals (CIs) were calculated for key performance metrics. All statistical analyses were performed with SPSS software (version 22.0.0, IBM Corp., Armonk, NY, USA), and a two-sided P < 0.05 was considered statistically significant. An overview of the study workflow is presented in Fig. 3.

### Role of the funding source

The funder of the study had no role in study design, data collection, data analysis, data interpretation, or writing of the report.

## Results

### Patients

This study finally included a total of 1004 patients with ISTs, including 723 in the internal dataset (yielding 4032 multi-sequence samples), 281 in the external dataset (yielding 981 samples). Each samples consisted of four aligned slices from different MRI sequences (sagittal T1W, T2W, T2FS, and axial T2W). As shown in Table 1, the age of patients range across the three cohorts was 5–89 years, with mean ages of 58 years (training and validation set), 59 years (internal test set), and 59 years (external test set), showing no significant differences among groups (P > 0.05). A higher prevalence of female patients was observed in our cohort, which is consistent with previous clinical reports indicating that meningiomas occur more frequently in females.^19^ Detailed numbers for each ISTs are presented in Supplemental Table S5.

**Table 1:** Characteristics of patients and IST lesions in primary and external sets.

Characteristic	Training set (n = 506)	Validation set (n = 73)	Internal test set (n = 144)	External test set (n = 281)	P
**Age (y)**^a^	58 ± 10 (6–89)	58 ± 10 (5–81)	59 ± 10 (5–85)	59 ± 8 (9–80)	0.332
**Sex**					0.982
Female	283 (55.9)	40 (54.8)	80 (55.6)	158 (56.2)	
Male	223 (44.1)	33 (45.2)	64 (44.4)	123 (43.8)	
**Pathology type**					0.999
Schwannoma	114 (22.5)	16 (21.9)	32 (22.2)	65 (23.1)	
Meningioma	110 (21.7)	16 (21.9)	32 (22.2)	61 (21.7)	
Astrocytoma	77 (15.2)	11 (15.1)	22 (15.3)	40 (14.2)	
Ependymoma	89 (17.6)	13 (17.8)	25 (17.4)	55 (19.6)	
Metastasis	116 (23.0)	17 (23.3)	33 (22.9)	60 (21.4)	
**Location**					0.633
C-spine	114 (22.6)	15 (20.5)	30 (20.8)	56 (19.9)	
T-spine	192 (37.9)	27 (37.0)	56 (38.9)	96 (34.2)	
l-spine	171 (33.8)	24 (32.9)	48 (33.3)	92 (32.7)	
S-spine	29 (5.7)	7 (9.6)	10 (7.0)	37 (13.2)	

Note: Unless otherwise stated, values are numbers of patients, with percentages in parentheses.

Abbreviation: IST, intraspinal tumor.

a Data are mean ± standard deviations, with the range in parentheses.

### Performance of ISMF-Net

Table 2 presents performance of ISMF-Net on validation, internal test, and external test sets for differentiating major types of ISTs. Average F1-score of 0.852 [0.818, 0.884], 0.843 [0.819, 0.867] and 0.825 [0.805, 0.844], micro-ACC of 0.859 [0.827, 0.889], 0.849 [0.826, 0.874] and 0.821 [0.801, 0.841], and AUC values of 0.922 [0.887, 0.957], 0.910 [0.880, 0.941] and 0.892 [0.860, 0.924] were obtained on the validation, internal test, and external test sets, respectively. These results demonstrate the model's stable performance across different datasets, highlighting its strong cross-center adaptability and generalization capability. Class-wise performance analysis revealed that ISMF-Net exhibited notable advantages in differentiation SCN and MNG with F1-score of 0.905 [0.879, 0.930]–0.940 [0.910, 0.968] and 0.863 [0.832, 0.893]–0.891 [0.836, 0.943] respectively. These results suggest that the model is highly effective in identifying these two common extramedullary tumors, which typically present with regular morphology, well-defined boundaries, and homogeneous spatial distribution. In contrast, the differentiation performance for AST and MET were relatively lower, with F1-score of 0.782 [0.745, 0.818]–0.790 [0.712, 0.862], 0.757 [0.724, 0.790]–0.804 [0.743, 0.858], respectively. ROC curves of ISMF-Net are shown in Supplementary Figure S7. The limited number of training samples for AST, an intramedullary tumor with low clinical incidence,^20^ may have constrained the model's learning capacity. Additionally, MET exhibits considerable heterogeneity in both morphology and anatomical location, making it more prone to misclassification. The confusion matrix of ISMF-Net on the validation, internal test, and external test sets (Supplementary Figure S8) further illustrate these misclassification patterns, showing that most errors occurred among AST, EPN, and MET. Comparative results with several state-of-the-art methods (Table 3) further confirm ISMF-Net's superior discriminative performance, achieving the highest micro-accuracy (0.849 [0.826, 0.874]) and AUC (0.910 [0.880, 0.941]). Detailed ROC curves, confusion matrix, and state-of-the-art experimental procedures are provided in the Supplementary Materials.

**Table 2:** Performance metrics of the ISMF-Net for differentiation of five types of ISTs.

	Precision	Recall	F1-score	AUC	Micro-ACC
**Validation average**	0.844 [0.812, 0.878]	0.868 [0.837, 0.899]	0.852 [0.818, 0.884]	0.922 [0.887, 0.957]	0.859 [0.827, 0.889]
Schwannomas	0.929 [0.887, 0.968]	0.952 [0.911, 0.984]	0.940 [0.910, 0.968]	0.961 [0.937, 0.986]	
Meningiomas	0.838 [0.760, 0.921]	0.950 [0.883, 1.000]	0.891 [0.836, 0.943]	0.960 [0.926, 0.995]	
Astrocytoma	0.758 [0.672, 0.853]	0.825 [0.719, 0.912]	0.790 [0.712, 0.862]	0.918 [0.891, 0.945]	
Ependymomas	0.778 [0.702, 0.857]	0.900 [0.829, 0.957]	0.834 [0.774, 0.889]	0.925 [0.882, 0.969]	
Metastasis	0.917 [0.861, 0.967]	0.715 [0.634, 0.789]	0.804 [0.743, 0.858]	0.845 [0.799, 0.891]	
**Internal test average**	0.835 [0.811, 0.859]	0.858 [0.834, 0.881]	0.843 [0.819, 0.867]	0.910 [0.880, 0.941]	0.849 [0.826, 0.874]
Schwannomas	0.928 [0.898, 0.958]	0.936 [0.903, 0.964]	0.932 [0.909, 0.954]	0.953 [0.935, 0.972]	
Meningiomas	0.852 [0.799, 0.907]	0.908 [0.850, 0.959]	0.879 [0.839, 0.918]	0.942 [0.912, 0.971]	
Astrocytoma	0.723 [0.660, 0.786]	0.868 [0.798, 0.930]	0.789 [0.737, 0.837]	0.909 [0.872, 0.946]	
Ependymomas	0.795 [0.739, 0.851]	0.857 [0.800, 0.907]	0.825 [0.780, 0.867]	0.907 [0.874, 0.941]	
Metastasis	0.878 [0.835, 0.917]	0.722 [0.665, 0.778]	0.792 [0.750, 0.831]	0.841 [0.808, 0.873]	
**External test average**	0.819 [0.800, 0.839]	0.840 [0.820, 0.858]	0.825 [0.805, 0.844]	0.892 [0.860, 0.924]	0.821 [0.801, 0.841]
Schwannomas	0.924 [0.894, 0.954]	0.887 [0.847, 0.923]	0.905 [0.879, 0.930]	0.934 [0.914, 0.955]	
Meningiomas	0.838 [0.799, 0.878]	0.888 [0.849, 0.924]	0.863 [0.832, 0.893]	0.925 [0.902, 0.948]	
Astrocytoma	0.691 [0.646, 0.736]	0.902 [0.855, 0.943]	0.782 [0.745, 0.818]	0.892 [0.836, 0.949]	
Ependymomas	0.807 [0.766, 0.849]	0.829 [0.780, 0.871]	0.818 [0.784, 0.850]	0.891 [0.858, 0.925]	
Metastasis	0.836 [0.800, 0.872]	0.692 [0.648, 0.737]	0.757 [0.724, 0.790]	0.818 [0.791, 0.845]	

Note: Data are presented as mean [95% confidence interval]. Average values represent macro-averages calculated across all classes. Abbreviations: ISTs, intraspinal tumors; Micro-ACC, micro-averaged accuracy; AUC, area under the receiver operating characteristic curve.

**Table 3:** Performance comparison with state-of-the-art models.

Method	Precision	Recall	F1-score	AUC	Micro-ACC
Resnet 50^16^	0.768 [0.748, 0.788]	0.789 [0.760, 0.818]	0.778 [0.748, 0.809]	0.853 [0.829, 0.876]	0.787 [0.758, 0.813]
NAMSTCD^21^	0.808 [0.780, 0.835]	0.832 [0.809, 0.855]	0.819 [0.781, 0.859]	0.887 [0.855, 0.920]	0.826 [0.800, 0.850]
TabNet^9^	0.710 [0.688, 0.732]	0.735 [0.714, 0.756]	0.722 [0.700, 0.745]	0.801 [0.766, 0.836]	0.742 [0.712, 0.770]
Optimized DenseNet^22^	0.793 [0.774, 0.821]	0.816 [0.778, 0.843]	0.803 [0.774, 0.830]	0.874 [0.842, 0.907]	0.813 [0.784, 0.839]
MR-Net^23^	0.780 [0.751, 0.819]	0.800 [0.777, 0.828]	0.790 [0.764, 0.820]	0.862 [0.829, 0.896]	0.799 [0.771, 0.824]
TransMed^24^	0.820 [0.793, 0.847]	0.845 [0.820, 0.871]	0.832 [0.804, 0.852]	0.898 [0.866, 0.930]	0.839 [0.803, 0.862]
ISMF-Net	0.835 [0.811, 0.859]	0.858 [0.834, 0.881]	0.843 [0.819, 0.867]	0.910 [0.880, 0.941]	0.849 [0.826, 0.874]

Note: Data are presented as mean [95% confidence interval]. Abbreviations: AUC, area under the receiver operating characteristic curve; Micro-ACC, micro-averaged accuracy.

**Fig. 4:**
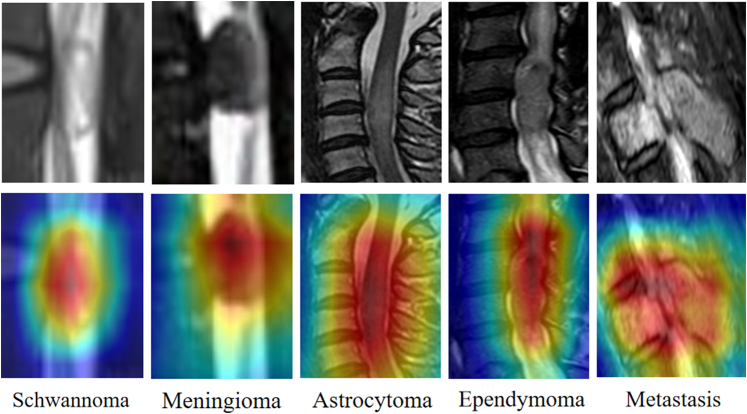
Grad-CAM visualizations of ISMF-Net across intraspinal tumor (IST) differentiation tasks.

Fig. 4 shows Grad-CAM visualizations of the ISMF-Net across ISTs differentiation tasks. It presents that SCN and MNG, the attention heatmaps are predominantly concentrated in the lateral spinal canal or epidural region, which aligns with their characteristic imaging manifestations. In contrast, for AST, due to their invasive margins, the heatmap distribution extends beyond the spinal canal boundaries, while for EPN, the activated regions are primarily localized within the spinal cord parenchyma, demonstrating the model's capability to effectively capture intramedullary morphological and signal characteristics. In the analysis of MET, the model consistently highlights the overall tumor extent and distribution patterns, reflecting its sensitivity in identifying the spatial heterogeneity of MET.

### Ablation study

The ablation study (Table 4) indicates the critical contributions of ISMF-Net's proposed modules and multimodal MRI sequences. In module ablation, consistent performance improvements were obtained with the incremental addition of GLAFM, SFM, MIM, SAMM, PAMM and clinical features. Incorporation of clinical features alone notably boosted Micro-ACC from 0.834 [0.793, 0.860] to 0.849 [0.826, 0.874] and F1-score from 0.833 [0.808, 0.855] to 0.843 [0.819, 0.867]. The complete ISMF-Net achieved the highest overall performance, demonstrating the synergistic effect of these components. Furthermore, sequence ablation analysis highlighted the indispensable and complementary value of each sagittal MRI sequence. While T1W and T2W sequences yielded a Micro-ACC of 0.774 [0.747, 0.803], the addition of T2-FS significantly improved Micro-ACC to 0.808 [0.783, 0.835] and F1-score to 0.801 [0.782, 0.821], suggesting its critical role in enhancing lesion detectability. The optimal performance of the full ISMF-Net with all sequences ultimately demonstrates that effective fusion of diverse multimodal imaging features is crucial for high-precision ISTs differentiation.

**Table 4:** Ablation study of different modules and sequences for ISMF-Net.

Method	Precision	Recall	F1-score	AUC	Micro-ACC
Baseline	0.770 [0.741, 0.801]	0.781 [0.752, 0.811]	0.775 [0.754, 0.796]	0.853 [0.816, 0.891]	0.783 [0.756, 0.809]
Baseline + GLAFM	0.787 [0.759, 0.816]	0.802 [0.774, 0.830]	0.794 [0.775, 0.815]	0.868 [0.832, 0.903]	0.798 [0.773, 0.825]
Baseline + GLAFM + SFM	0.808 [0.781, 0.836]	0.825 [0.798, 0.852]	0.816 [0.797, 0.836]	0.882 [0.849, 0.917]	0.818 [0.791, 0.844]
Baseline + GLAFM + SFM + MIM	0.811 [0.783, 0.838]	0.832 [0.806, 0.859]	0.821 [0.809, 0.847]	0.887 [0.852, 0.920]	0.821 [0.789, 0.846]
Baseline + GLAFM + SFM + MIM + SAMM	0.818 [0.791, 0.847]	0.842 [0.816, 0.868]	0.829 [0.802, 0.853]	0.893 [0.860, 0.926]	0.832 [0.803, 0.859]
Baseline + GLAFM + SFM + MIM + SAMM + PAMM	0.821 [0.795, 0.848]	0.845 [0.819, 0.871]	0.833 [0.808, 0.855]	0.896 [0.863, 0.928]	0.834 [0.793, 0.860]
ISMF-Net (Baseline + GLAFM + SFM + MIM + SAMM + PAMM + clinical features)	0.835 [0.811, 0.859]	0.858 [0.834, 0.881]	0.843 [0.819, 0.867]	0.910 [0.880, 0.941]	0.849 [0.826, 0.874]
T1 + T2 (sagittal)	0.763 [0.713, 0.793]	0.779 [0.749, 0.809]	0.770 [0.750, 0.792]	0.844 [0.806, 0.883]	0.774 [0.747, 0.803]
T1 + T2 + T2-fs (sagittal)	0.791 [0.763, 0.820]	0.812 [0.784, 0.839]	0.801 [0.782, 0.821]	0.875 [0.840, 0.910]	0.808 [0.783, 0.835]
ISMF-Net (sagittal + axial)	0.835 [0.811, 0.859]	0.858 [0.834, 0.881]	0.843 [0.819, 0.867]	0.910 [0.880, 0.941]	0.849 [0.826, 0.874]

Note: Data are presented as mean [95% confidence interval]. Abbreviations: GLAFM, global-local attention fusion module; SFM, sequence fusion module; MIM, multi-plane fusion module; SAMM, sequence-aware anomaly matrix module; PAMM, plane-aware anomaly matrix module; AUC, area under the receiver operating characteristic curve; micro-ACC, micro-averaged accuracy; T1, T1-weighted imaging; T2, T2-weighted imaging; T2-fs, T2 fat-suppressed imaging.

### Observer study

**Fig. 5:**
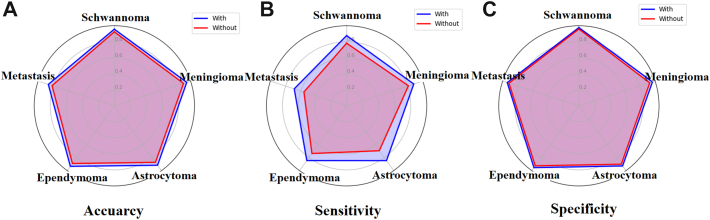
Performance of radiologists in differentiating five types of intraspinal tumors (ISTs) with and without the assistance of ISMF-Net in external test set. Each radar chart represents one of three metrics: accuracy (A), sensitivity (B), and specificity (C).

As shown in Supplementary Table S7, without model assistance, the average diagnostic accuracy, sensitivity, and specificity for all ISTs were 0.882 [0.879, 0.886], 0.719 [0.710, 0.728], and 0.926 [0.924, 0.929], respectively. The radiologists demonstrated high accuracy in diagnosing SCN, MNG, and EPN, especially among senior radiologists. However, for MET and AST, diagnostic accuracy was low, posing significant challenges, especially for junior radiologists. With model assistance, the average diagnostic accuracy, sensitivity, and specificity for all ISTs improved to 0.925 [0.922, 0.928], 0.827 [0.820, 0.834], and 0.953 [0.951, 0.955], indicating a substantial increase in diagnostic performance. Fig. 5 illustrates the overall improvement in radiologists’ diagnostic performance with ISMF-net assistance. The most notable improvements were observed for AST and MET, with accuracy increasing to 0.917 [0.912, 0.922] and 0.866 [0.860, 0.873], sensitivity to 0.846 [0.826, 0.865] and 0.687 [0.670, 0.704], and specificity to 0.928 [0.923, 0.934] and 0.940 [0.935, 0.946], respectively. These results suggest that the model may offer valuable assistance in the differentiation of ambiguous lesions.

**Fig. 6:**
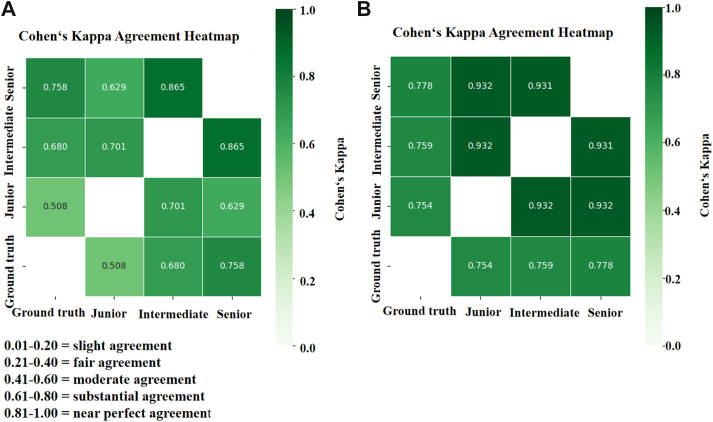
Cohen's kappa heatmaps showing agreements among radiologists and the ground truth: without (A) and with (B) model assistance.

As shown in Table 5, for all five types of ISTs, the model yielded the most substantial performance enhancements among junior radiologists, with improvements of 7.9%, 20.1%, and 4.9% in accuracy, sensitivity, and specificity, respectively. Intermediate radiologist also benefited notably (2.5%, 6.0%, and 1.5%), while senior radiologists showed slight improvements. Additionally, Cohen's Kappa analysis (Fig. 6) further revealed that model assistance enhanced diagnostic consistency across all radiologists of different levels of experience. For junior radiologists, agreement with intermediate- and senior-level radiologists increased by 23.1% and 30.3%, respectively. With model assistance, junior radiologists achieved diagnostic performance comparable to that of intermediate radiologist, and approached senior radiologist performance in diagnosing SCN and MNG. This indicates the ISMF-Net may provide an effective approach to addressing diagnostic challenges in settings with limited medical resources or specialist availability (Detailed diagnostic results of each radiologist and the consistency analysis are provided in the Supplement Materials). Supplementary Figure S12 presents eight representative ISTs from the external test set, highlighting diagnostic differences between the model and radiologists of different levels of experience. Specifically, in cases with ambiguous imaging features or diagnostically challenging complexity, the model outperformed junior and intermediate radiologists and demonstrated diagnostic accuracy comparable to that of senior radiologist. However, in the diagnosis of complex MET, senior radiologists exhibited greater stability and consistency.

**Table 5:** Comparison of the radiologist group's average performance with and without DL model assistance in the external test set.

	Accuracy		Sensitivity		Specificity	
	without	with	without	with	without	with
**Senior average**	0.925 [0.919, 0.931]	0.931 [0.926, 0.936]	0.827 [0.813, 0.841]	0.843 [0.830, 0.856]	0.953 [0.950, 0.957]	0.957 [0.953, 0.960]
Schwannomas	0.956 [0.949, 0.964]	0.961 [0.954, 0.968]	0.889 [0.861, 0.914]	0.896 [0.870, 0.922]	0.973 [0.966, 0.980]	0.977 [0.971, 0.983]
Meningiomas	0.951 [0.943, 0.959]	0.954 [0.946, 0.961]	0.888 [0.861, 0.914]	0.894 [0.869, 0.920]	0.966 [0.958, 0.972]	0.967 [0.960, 0.974]
Astrocytoma	0.919 [0.909, 0.929]	0.926 [0.917, 0.935]	0.829 [0.790, 0.865]	0.860 [0.824, 0.894]	0.934 [0.924, 0.943]	0.936 [0.927, 0.946]
Ependymomas	0.935 [0.925, 0.943]	0.942 [0.934, 0.951]	0.839 [0.806, 0.869]	0.865 [0.835, 0.894]	0.957 [0.948, 0.965]	0.960 [0.952, 0.968]
Metastasis	0.866 [0.853, 0.877]	0.872 [0.860, 0.884]	0.693 [0.660, 0.724]	0.700 [0.668, 0.732]	0.938 [0.926, 0.948]	0.943 [0.933, 0.954]
**Intermediate average**	0.898 [0.892, 0.905]	0.923 [0.917, 0.928]	0.761 [0.745, 0.777]	0.821 [0.807, 0.835]	0.936 [0.932, 0.940]	0.951 [0.948, 0.955]
Schwannomas	0.931 [0.923, 0.941]	0.957 [0.949, 0.964]	0.801 [0.766, 0.834]	0.878 [0.850, 0.903]	0.963 [0.956, 0.971]	0.976 [0.969, 0.982]
Meningiomas	0.925 [0.916, 0.935]	0.950 [0.942, 0.958]	0.827 [0.793, 0.859]	0.877 [0.847, 0.904]	0.947 [0.938, 0.956]	0.966 [0.959, 0.973]
Astrocytoma	0.899 [0.889, 0.910]	0.917 [0.907, 0.926]	0.777 [0.736, 0.819]	0.829 [0.790, 0.864]	0.919 [0.908, 0.930]	0.931 [0.921, 0.941]
Ependymomas	0.907 [0.897, 0.917]	0.928 [0.919, 0.937]	0.772 [0.736, 0.806]	0.837 [0.804, 0.867]	0.939 [0.929, 0.948]	0.950 [0.941, 0.958]
Metastasis	0.828 [0.815, 0.842]	0.862 [0.850, 0.874]	0.627 [0.594, 0.660]	0.685 [0.653, 0.717]	0.912 [0.900, 0.924]	0.935 [0.924, 0.946]
**Junior average**	0.843 [0.837, 0.849]	0.922 [0.917, 0.927]	0.620 [0.605, 0.635]	0.821 [0.801, 0.832]	0.902 [0.899, 0.906]	0.951 [0.948, 0.958]
Schwannomas	0.899 [0.890, 0.907]	0.955 [0.949, 0.961]	0.700 [0.669, 0.731]	0.864 [0.839, 0.887]	0.947 [0.940, 0.955]	0.977 [0.972, 0.982]
Meningiomas	0.867 [0.857, 0.877]	0.947 [0.941, 0.954]	0.749 [0.717, 0.780]	0.875 [0.851, 0.897]	0.893 [0.883, 0.904]	0.963 [0.957, 0.970]
Astrocytoma	0.822 [0.811, 0.833]	0.911 [0.902, 0.920]	0.546 [0.506, 0.587]	0.848 [0.819, 0.876]	0.867 [0.856, 0.878]	0.921 [0.913, 0.930]
Ependymomas	0.849 [0.839, 0.859]	0.932 [0.923, 0.939]	0.644 [0.611, 0.678]	0.838 [0.811, 0.863]	0.897 [0.887, 0.907]	0.953 [0.946, 0.960]
Metastasis	0.777 [0.766, 0.787]	0.865 [0.856, 0.875]	0.461 [0.434, 0.488]	0.680 [0.654, 0.706]	0.908 [0.897, 0.918]	0.942 [0.933, 0.950]

Note: Data are presented as mean [95% confidence interval]. Two senior radiologists (Senior 1: 30 and Senior 2: 20 years of experience), two intermediate radiologists (intermediate 1:12 and intermediate 2: 11 years of experience), and three junior radiologists (junior 1:6, junior 2:4, and junior 3:1 year of experience, respectively), average = macro-average.

MRI features exhibit multi-sequence and multi-plane characteristics, with each modality providing complementary diagnostic information.^25^ Typically, each type of ISTs typically corresponds to a preferred sequence or imaging plane, consistent with radiologists' clinical diagnostic practices. For example, in SCN diagnosis, radiologists prioritized T2-weighted images (90%), T2-FS (85%), and T1-weighted images (50%). We assessed the correlation between the model's F1-scores and the clinically preferred imaging sequences. Among the five types of ISTs, SCN, MNG, AST, and EPN exhibited strong positive correlations (r = 0.994, 0.927, 0.957, and 0.989, respectively). These findings suggest a high degree of alignment between the model's attention patterns and radiologists' diagnostic focus, supporting the clinical relevance and scientific soundness of our approach. In contrast, MET showed a weaker correlation (r = 0.640), likely reflecting the balanced diagnostic contribution of all three sequences. Overall, the ISMF-Net effectively captures clinically relevant features and demonstrates expert-level performance in sequence-specific interpretation. Additionally, analysis of plane preferences revealed no significant differences (Detailed analysis is provided in the Supplement Materials).

## Discussion

The diagnosis of ISTs remains challenging due to their heterogeneous lesion types, indistinct margins, and complex anatomical variability, leading to subjective interpretation and misclassification in clinical assessments.^7^ Deep learning has shown potential to improve diagnostic accuracy for ISTs.^26^ However, existing DL methods generally do not adequately model the elongated anatomical structure of the spinal canal and often treat multimodal MRI sequences and clinical data with simple concatenation or augmentation approaches, neglecting the complementary of tissue contrast, boundary clarity, and lesion sensitivity.^8^, ^9^, ^10^, ^11^, ^12^ To address these limitations, this study proposes ISMF-Net, which integrates complementary information from four MRI sequences and clinical data through a cross-modal feature fusion strategy, thereby improving the model's generalization and diagnostic performance, with Micro-ACC of 0.859 [0.827, 0.889], 0.849 [0.826, 0.874], and 0.821 [0.801, 0.841] on the validation, internal test, and external test sets, respectively. Grad-CAM visualizations show that the model consistently focuses on the tumor core and adjacent critical anatomical structures during prediction, demonstrating robust localization and discriminative capacity across classes.

This performance is attributed to the innovative modular architecture of ISMF-Net. A adapted ResNet-Transformer dual-encoder architecture, incorporating DSConv^13^ and DCN,^17^ was employed to more effectively extract features from slender and non-rigid lesions, offering greater flexibility and efficiency than ResNet^16^ models in handling complex and variable ISTs. To enhance the integration of local texture and global context, ISMF-Net incorporates the GLAFM, which employs parallel attention mechanisms to address the limitations of sequential attention and achieve more effective feature fusion. Compared to single-sequence fusion methods such as CBAM,^27^ GLAFM demonstrates superior lesion representation, as evidenced by consistent F1-score improvements in ablation and comparative studies (Supplementary Table S6). The SFM, together with the SAMM, adopts a data-driven weighting strategy to effectively integrate complementary diagnostic information from T1W, T2W, and T2-FS sequences. Compared to conventional fusion approaches such as direct concatenation or single-sequence attention modules,^8^ this design offers improved efficiency and adaptability in extracting features from complex ISTs. Furthermore, to address the limitations of single-plane feature modeling, ISMF-Net incorporates a MIM with a PAMM, which enhances the capture of anatomical variations across sagittal and axial views. Compared to single-plane-based models,^8^ MIM enables richer spatial representation from multiple perspectives. Ablation studies confirm its effectiveness, with significant drops in precision and recall observed when MIM is removed. Additionally, PAMM addresses the limitations of traditional single-stage feature fusion methods,^28^ improving the accuracy and effectiveness of feature fusion through a two-stage mechanism.

The observer study demonstrated that ISMF-Net significantly enhanced diagnostic performance across radiologists of varying experience levels, increasing diagnostic accuracy, sensitivity, and specificity by 4.3%, 10.8%, and 2.7%, respectively, and improving inter-rater agreement (Cohen's kappa). With model assistance, junior radiologists achieved diagnostic performance equivalent to that of intermediate radiologists, and demonstrated diagnostic performance comparable to that of senior radiologists in SCN and MNG differentiation, reducing misdiagnosis rates. However, in MET cases, junior radiologists made errors despite initial correct judgments, likely due to over-reliance on the model. Nevertheless, junior radiologists benefited most, indicating the model’ s effectiveness in improving their diagnostic skills. In contrast, senior radiologists showed greater stability and accuracy, with the model providing additional support, further validating its robustness. These findings suggest that ISMF-Net may contribute to reducing inter-observer variability by promoting more consistent feature extraction and integration, which could in turn minimize the effect of individual subjective tendencies in image interpretation. Preference analysis confirmed that the model’ s predictions aligned with clinical observations, enhancing interpretability and validating clinical correlations. Thus, ISMF-Net effectively assists radiologists in making accurate diagnoses, addresses the imbalance in medical resource distribution, and helps local radiologists optimize limited resources for improved patient care quality. Compared to radiologists, the model showed high accuracy and stability in eight challenging cases. However, for patients 6, 7, and 8, all involving MET, the model's performance differed significantly from senior radiologists. This may be due to the limited number of complex cases in the dataset. Future work should expand the dataset with more challenging cases to improve model robustness.

This study has limitations. First, the sample size, particularly for low-incidence subtypes of ISTs such as AST and EPN, is relatively limited and warrants further validation in larger multi-center cohorts. Second, the reliance on complete multi-modal MRI sequences poses challenges for applicability in clinical practice, where missing sequences are common. Furthermore, while the proposed framework achieves high diagnostic performance, its computational demands may limit practical deployment in resource-constrained settings, highlighting the need for optimization to balance efficiency and accuracy. Therefore, future work will focus on optimizing the model architecture, such as introducing lightweight network designs, to enhance generalizability and computational efficiency while maintaining diagnostic accuracy. Further investigation and prospective validation on datasets with diverse imaging protocols and population characteristics will also be conducted to ensure broader clinical applicability.

In conclusion, this study proposes a multi-modal MRI-based ISMF-Net incorporating cross-modal feature fusion mechanism, and achieved accurate and efficient ISTs diagnosis. The ISMF-Net shows promise as a non-invasive diagnostic tool, potentially enhancing diagnostic accuracy and supporting improved clinical decision-making in ISTs management.

## Contributors

X.R.J. and Q.H.Z. contributed to study concepts and manuscript preparation. X.R.J., Q.H.Z. and J.X.Y. contributed to study design. Y.Y.S., Q.H.Z., C.N.Y., Q.G., and X.Q.C. contributed to data acquisition. Q.H.Z., C.N.Y. and J.X.Y. contributed to quality control of data and algorithms. F.Y.Z, Q.H.Z. and B.H. contributed to data analysis and interpretation. H.H.C. contributed to statistical analysis. X.R.J. and H.H.C contributed to manuscript review. X.R.J. and Q.H.Z. had full access to all the data in the study and verified the underlying data. All authors read and approved the final manuscript.

## Data sharing statement

The data and code that support the findings of this study are available from the corresponding author upon reasonable request and with permission from the institutional ethics committees. Individual patient imaging data cannot be shared publicly due to privacy and data protection regulations.

## Declaration of interests

The authors declare no competing interests.
