# Supplementary material for: Development and validation of a multi-modal MRI-based deep learning framework for differentiation of intraspinal tumors (ISMF-Net)
Source: eClinicalMedicine. 2025 Nov 13;90:103636. doi: 10.1016/j.eclinm.2025.103636 (PMC12663647; doi:10.1016/j.eclinm.2025.103636)
Supplement: Supplementary Files [file mmc1.docx]

[Methods： 2](#_Toc211626307)

[Patients 2](#_Toc211626308)

[MRI Image Preprocessing 2](#_Toc211626309)

[Methodology 4](#_Toc211626310)

[Clinical feature embedding 4](#_Toc211626311)

[Design of Feature Extraction Module 4](#_Toc211626312)

[GLAFM 5](#_Toc211626313)

[SFM 6](#_Toc211626314)

[MIM 7](#_Toc211626315)

[SAMM and PAMM 7](#_Toc211626316)

[Performance Metrics 8](#_Toc211626317)

[Experimental 8](#_Toc211626318)

[Results 9](#_Toc211626319)

[Patients 9](#_Toc211626320)

[Performance of the ISMF-Net 9](#_Toc211626321)

[State-of-the-art methods 10](#_Toc211626322)

[Ablation study 10](#_Toc211626323)

[Observer study 10](#_Toc211626324)

[Comparison of the radiologist group's average performance with and without ISMF-Net assistance. 10](#_Toc211626325)

[Analysis of sequence preference between radiologists and ISMF-Net. 13](#_Toc211626326)

[Plan preference analysis 14](#_Toc211626327)

[Comparative case analysis of seven radiologists and ISMF-Net 15](#_Toc211626328)

[References 15](#_Toc211626329)

Methods：

Patients

Each sample consisted of four spatially aligned slices from different MRI sequences (sagittal T1W, sagittal T2W, sagittal T2-FS, and axial T2W). Table S1 summarizes the number of samples obtained from each of the three centers.

**Table S1. Distribution of multi-sequence slice samples from each center.**

|  | **Center 1** | **Center 2** | **Center 3** |
| --- | --- | --- | --- |
| Total | 4032 | 426 | 555 |
| Schwannomas | 959 | 130 | 144 |
| Meningiomas | 693 | 116 | 135 |
| Astrocytoma | 565 | 82 | 111 |
| Ependymomas | 643 | 142 | 121 |
| Metastasis | 1172 | 126 | 280 |

Note: Each training group corresponds to four MR sequences (sagittal T1, sagittal T2, sagittal T2-FS, axial T2) from the same slice of a patient.

MRI Image Preprocessing

Intraspinal tumors (ISTs) often represent small targets within large magnetic resonance imaging (MRI) scans, which can lead to considerable computational inefficiency, this study employs YOLOv9 for ISTs identification due to its robust performance in small object detection. The model introduced a modified YOLOv9^(1)^ architecture, specifically by replacing traditional convolutions with Dynamic Snake Convolutions (DSConv)^(2)^ in the RepNCSPELAN4_DSC module of the backbone (Figure S1). This adaptation enables more precise feature capture by conforming to the intricate morphology of the spinal canal. Post-detection, images are cropped based on identified coordinates. All cropped images undergo physician review, with erroneous detection or incorrect crops subjected to radiologist-guided re-cropping.

The YOLOv9 model was trained on annotated MRI data collected at Center 1 between April 2014 and March 2025 (n = 459 patients, 8,672 slices). Lesion locations were annotated as bounding boxes by two board-certified radiologists with reference to pathological findings. The dataset was randomly divided into training and testing sets in a 9:1 ratio (Table S2). Training was conducted with the Adam optimizer (initial learning rate = 0.001, batch size = 16) using a composite loss function consisting of CIoU loss for bounding box regression and binary cross-entropy loss for classification. Early stopping with a patience of 100 epochs was applied, and the model weights with the lowest validation loss were selected for evaluation. Inference was performed with a confidence threshold of 0.25 and an NMS IoU threshold of 0.5. Based on this trained model, newly collected cases from Centers 2 and 3 were directly subjected to inference to automatically generate lesion bounding boxes.

In the present study, additional newly collected cases were further included, on which the trained YOLOv9 model was directly applied to automatically generate lesion bounding boxes. Following detection, bounding box coordinates were extended by 40 pixels in all directions for cropping to preserve perilesional context. All cropped ROIs were subsequently reviewed by radiologists, and any erroneous detections or incorrect crops were corrected manually. This workflow allowed us to substantially reduce the manual annotation burden while ensuring the accuracy of lesion localization for downstream classification tasks. In the testing set, the YOLOv9 achieved a precision of 0.855, recall of 0.887, and F1-score of 0.870, with a mean average precision (mAP) of 0.883.


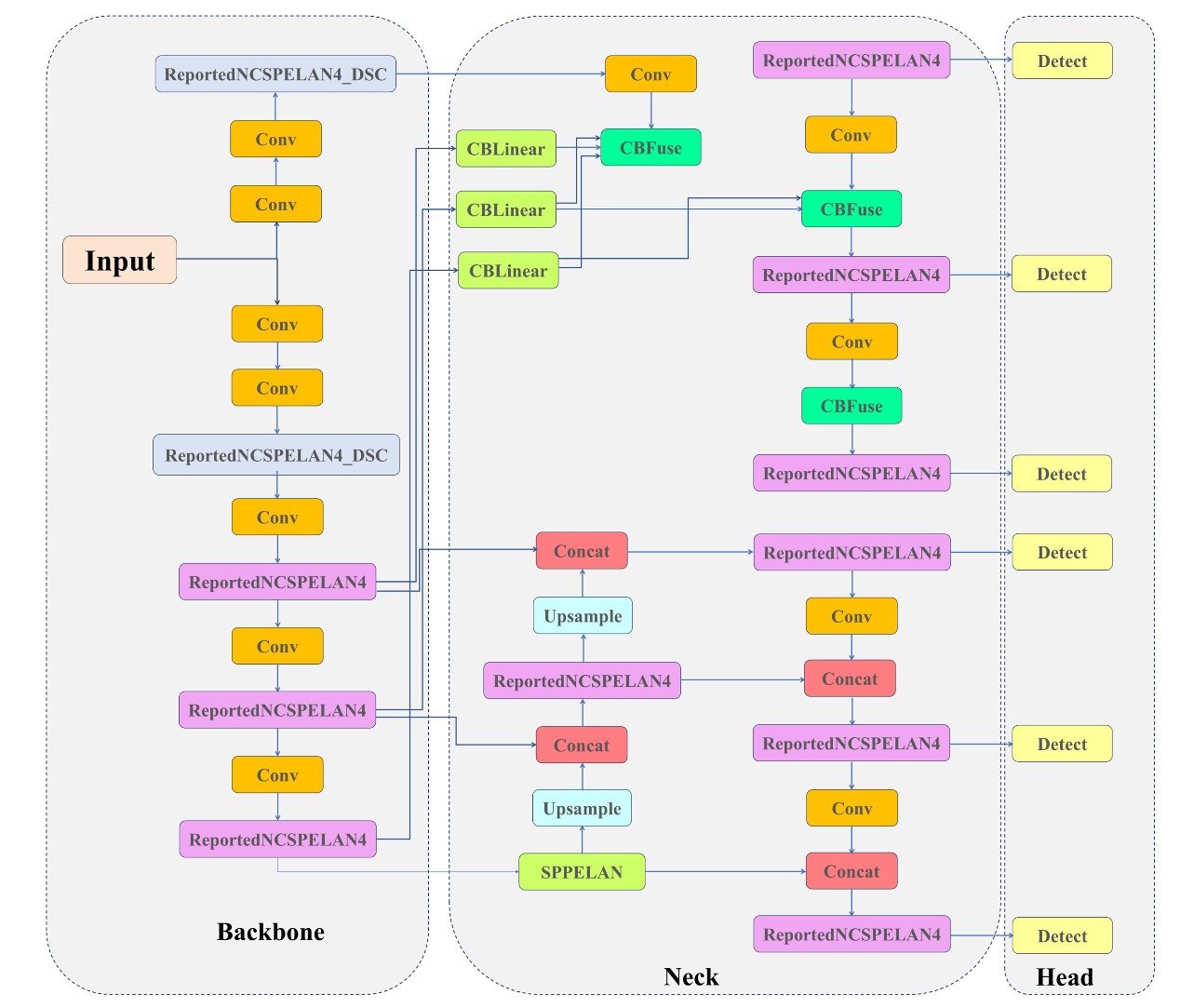


**Figure S1**. Architecture of the modified YOLOv9 network.

**Table S2. Distribution of samples across training and test sets.**

|  | **Training** | **Testing** |
| --- | --- | --- |
| Total | 7805 | 867 |
| Schwannomas | 1948 | 216 |
| Meningiomas | 1638 | 182 |
| Astrocytoma | 907 | 101 |
| Ependymomas | 1238 | 138 |
| Metastasis | 2084 | 232 |

To address potential class imbalance and prevent model bias towards prevalent categories, we implemented the image-level data augmentation strategy for the training set. For over-represented classes such as schwannomas (SCN), meningiomas (MNG), we applied minor augmentation through rotations, translations, and cropping. Conversely, for under-represented classes, specifically astrocytoma (AST) and ependymomas (EPN), we performed more substantial augmentation using the same techniques. This approach aims to balance the dataset distribution and enhance model generalization across all tumor types.

Specifically, half of the 583 schwannoma images and two-thirds of the 513 meningioma images were randomly selected, whereas all AST and ependymoma samples were augmented once, and MET cases were not expanded. The training set size increased from 2,716 to 4,170 images. Data augmentation was performed under the principle of preserving anatomical semantics. Mild spatial transformations and intensity perturbations were randomly combined, including translation up to 10% of edge length, rotation within ±10°, isotropic scaling between 0.9 and 1.1, random cropping to the network input size with up to 10% offset, horizontal flipping, brightness adjustment within ±10%, contrast adjustment between 0.9 and 1.1, gamma correction between 0.9 and 1.1, Gaussian noise with σ ≤ 0.03 relative to the normalized dynamic range, and motion or mean blurring with kernel size of 3 to 5 pixels. All transformations were sampled online and applied independently to the selected samples. The detailed changes in the number of training image groups before and after augmentation are presented in Table S3, and a comparison of validation set performance before and after augmentation is provided in Table S4.

**Table S3. Comparison of multi-sequence slice sample distribution in the training set without and with data augmentation.**

|  | **Training set(without)** | **Training set(with)** |
| --- | --- | --- |
| Total | 2716 | 4170 |
| Schwannomas | 583 | 875 |
| Meningiomas | 513 | 855 |
| Astrocytoma | 387 | 774 |
| Ependymomas | 433 | 866 |
| Metastasis | 800 | 800 |

Note: Each sample consisted of four aligned slices from different MRI sequences (sagittal T1W, T2W, T2FS, and axial T2W).

Table S4 demonstrates the effect of data augmentation on ISMF-Net where the F1-scores for AST and EPN increased after augmentation of the minority classes.

**Table S4. Comparison of validation set performance without and with data augmentation for ISMF-Net.**

|  | **Precision** | **Recall** | **F1-score** | **AUC** | **Micro-ACC** |
| --- | --- | --- | --- | --- | --- |
| **Validation Average (without)** | 0.837 [0.804, 0.871] | 0.861 [0.827, 0.892] | 0.844 [0.810, 0.877] | 0.912 [0.870,0.955] | 0.853 [0.820, 0.883] |
| Schwannomas | 0.921 [0.877, 0.965] | 0.944 [0.903, 0.984] | 0.932 [0.899, 0.964] |  |  |
| Meningiomas | 0.836 [0.757, 0.918] | 0.933 [0.867, 0.983] | 0.882 [0.824, 0.936] |  |  |
| Astrocytoma | 0.734 [0.644, 0.833] | 0.825 [0.719, 0.912] | 0.777 [0.698, 0.852] |  |  |
| Ependymomas | 0.775 [0.701, 0.857] | 0.886 [0.800, 0.957] | 0.827 [0.765, 0.886] |  |  |
| Metastasis | 0.917 [0.863, 0.967] | 0.715 [0.634, 0.789] | 0.804 [0.744, 0.857] |  |  |
| **Validation Average (with)** | 0.844 [0.812, 0.878] | 0.868 [0.837, 0.899] | 0.852 [0.818, 0.884] | 0.922[0.887, 0.957] | 0.859 [0.827, 0.889] |
| Schwannomas | 0.929 [0.887, 0.968] | 0.952 [0.911, 0.984] | 0.940 [0.910, 0.968] |  |  |
| Meningiomas | 0.838 [0.760, 0.921] | 0.950 [0.883, 1.000] | 0.891 [0.836, 0.943] |  |  |
| Astrocytoma | 0.758 [0.672, 0.853] | 0.825 [0.719, 0.912] | 0.790 [0.712, 0.862] |  |  |
| Ependymomas | 0.778 [0.702, 0.857] | 0.900 [0.829, 0.957] | 0.834 [0.774, 0.889] |  |  |
| Metastasis | 0.917 [0.861, 0.967] | 0.715 [0.634, 0.789] | 0.804 [0.743, 0.858] |  |  |

Note: Data are presented as mean [95% confidence interval]. The "Average" values are macro-average.

Methodology

Clinical feature embedding

Clinical information, including sex, age, and tumor location, was collected for each patient. Sex and tumor location were encoded using one-hot encoding, while age was normalized, resulting in a clinical feature vector. This vector was then concatenated with the features extracted individually from T1, T2, T2-FS, and axial T2W sequences, producing Ft1, Ft2 and Ft3, which were subsequently used for following feature fusion.​

Design of Feature Extraction Module

Figure S2 presents the architectural design for the dual extraction module in Figure 3A. For each MRI sequence in the sagittal and axial planes, features were independently extracted based on two parallel modules: a global feature branch and a local feature branch (Figure S2A). Global features were obtained using a stacked Transformer blocks^(3)^ architecture, in which the input images were first embedded into patch tokens and subsequently processed through four Transformer^(3)^ blocks to capture long-range contextual dependencies. The local feature branch was based on an Adaptive ResNet structure (Figure S2B). To better model the complex and anisotropic anatomical characteristics of the spinal canal, the standard Conv2D layers in the second and third blocks were replaced with Dynamic Snake Convolution (DSConv) ^(2)^and Deformable Convolution Network (DCN)^(4)^, which facilitate direction-aware feature extraction and improve the network’s capacity to capture subtle boundary variations and irregular morphological deformations. This design promotes both boundary-preserving feature representation and hierarchical context modeling. All extracted features are ultimately fed into the GLAFM module for cross-branch and cross-scale fusion.

**
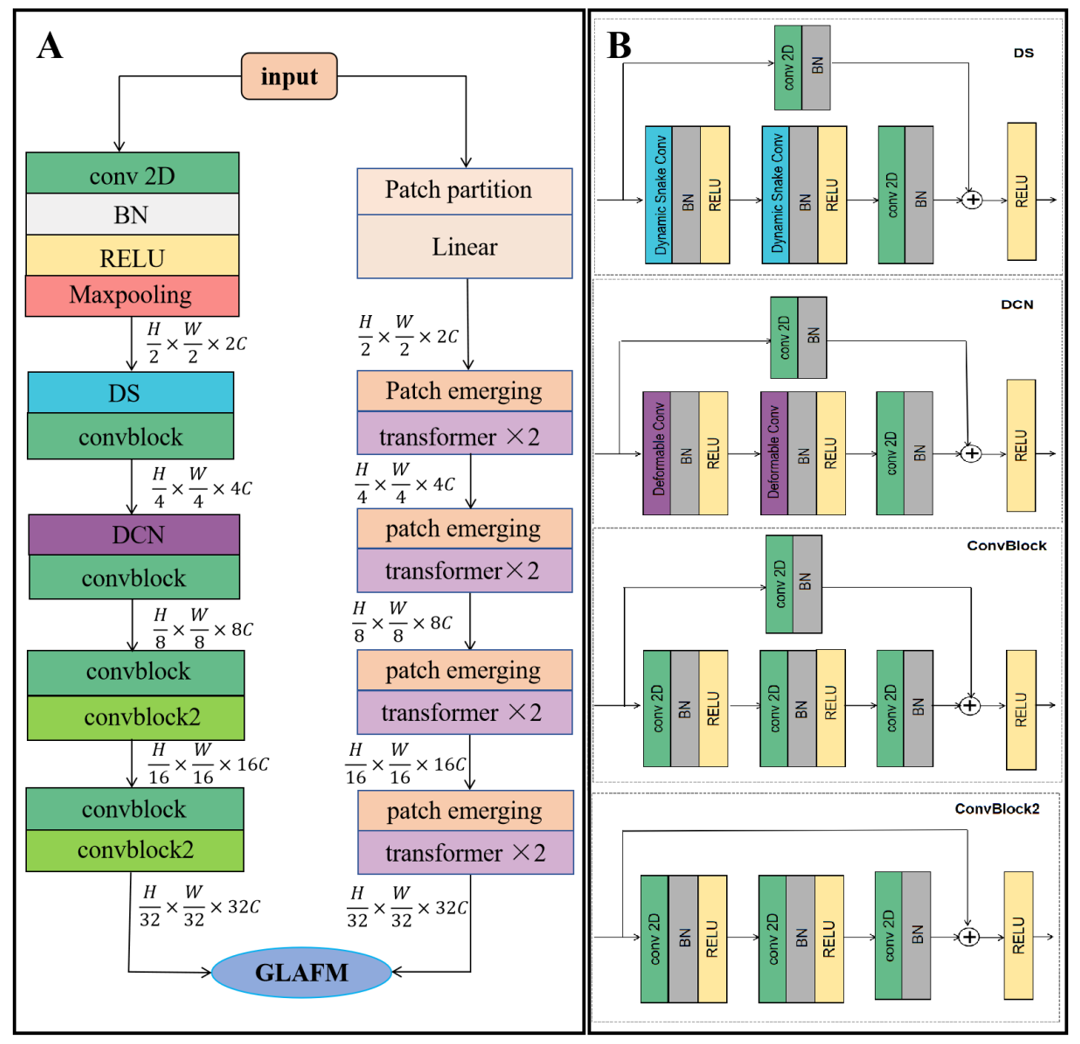
**

**Figure S2**. Overview of the proposed dual-branch feature extraction architecture. (A) Parallel CNN–Transformer pathways with progressive down sampling and modality-specific encoding. (B) Details of the internal structures of convolutional blocks, including Dynamic Snake Convolution (DSConv), Deformable Convolution Network (DCN), and standard residual blocks.

### **GLAFM**

The Global-Local Attention Fusion Module (GLAFM) (Figure S3) integrates global and local features as inputs to enhance the overall feature representation capability. In the global branch, a channel-first attention mechanism is employed to enhance channel-wise information, whereas the local branch is designed to capture spatial dependencies. Both branches utilize cascaded Channel Attention (CA) and Spatial Attention (SA) modules, albeit in reverse order, to emphasize different aspects of the features. Input global features are first processed by a Channel Attention (CA) module, which applies both average and max pooling, followed by a shared MLP to generate intermediate representations. The CA mechanism is defined as:$\mathrm{CA}\left( z \right)=\sigma\left( \text{α}\mathrm{MLP}\left( \mathrm{AvgPool}\left( z \right) \right)+\left( 1-\text{α} \right)\mathrm{MLP}\left( \mathrm{MaxPool}\left( z \right) \right) \right)\cdot z$, where AvgPool and MaxPool denote average and max pooling operations, respectively. These are linearly transformed and aggregated to adapt to tumor heterogeneity across subregions. The resulting weights are activated through a sigmoid function and applied to the input features for channel enhancement. A subsequent 1×1 convolution generates gating weights to balance channel and spatial attention, serving as input to a Spatial Attention (SA) module. The SA mechanism is expressed as:$\mathrm{SA}\left( z \right)=\sigma\left( f^{7\cdot7}\left( \left[ AvgPool\left( z \right);MaxPool\left( z \right) \right] \right) \right)\cdot z$ where $f^{7\cdot7}$is a learned transformation applied to the pooled features. The output is then concatenated with the CA features from the local branch. In contrast, the local branch emphasizes spatial dependencies. Input features first undergo a 1×1 convolution for channel adjustment, followed by an SA module and a CA module. Spatial attention weights are obtained via average pooling and two fully connected layers, then applied to the input features. Gating weights are similarly generated to refine attention contribution. The outputs from both branches are then fused as follows: $z_{1}=CA\left( z_{\mathrm{global}} \right)\cdot z_{\mathrm{global}}$, $z_{2}=\beta_{1}\mathrm{SA}\left( z_{1} \right)\cdot z_{1}, z_{3}=SA\left( z_{\mathrm{local}} \right)\cdot z_{\mathrm{local}}$, $z_{4}=\beta_{2}CA\left( z_{3} \right)\cdot z_{3}, z_{c}=cat(add\left( z_{3},z_{2} \right),cat(z_{1},z_{4}))$. These outputs from both branches are concatenated and fused through a 1×1 convolution and a CNN-based reduction module, resulting in a refined feature representation:$z_{\mathrm{out}}=MLP(\sigma\left( Conv\left( Flatten\left( z_{c} \right) \right) \right)$. This refined representation effectively balances global context with local details, enhancing the overall feature representation.


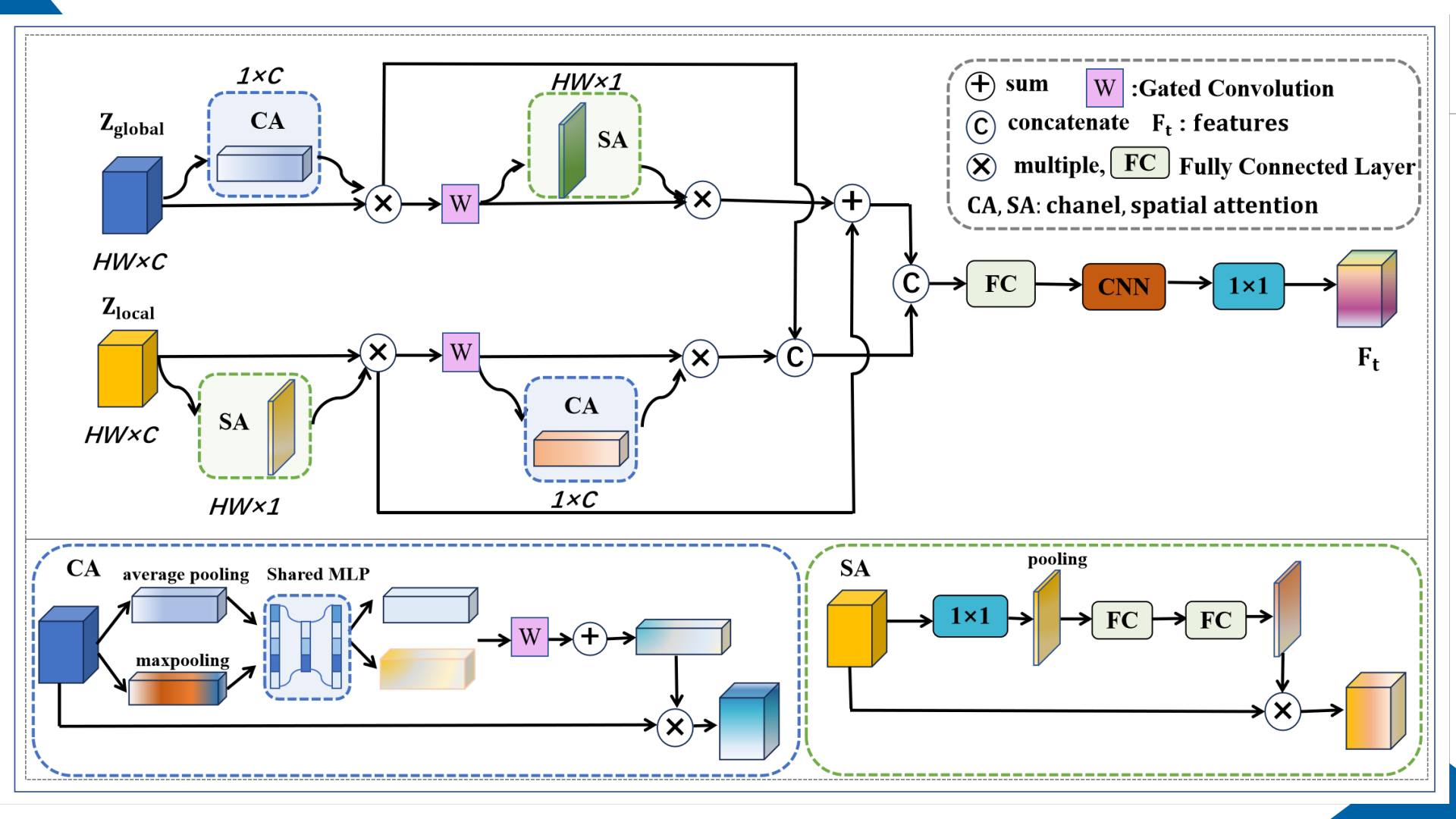


**Figure S3**. Overview of the proposed Global-Local Attention Fusion Module (GLAFM) architecture.

### **SFM**

Figure S4 presents the proposed Sequence Fusion Module (SFM), which aims to model the complementary information across multi-sequence MRI (T1, T2, T2-FS) and extract sequence-specific features for ISTs differentiation. Each sequence is treated as the primary modality in turn, with the remaining two as vice modalities. Using dot-product cross-attention, the primary modality serves as the query (Q), while the secondary modalities provide the keys and values (K, V). The attention is computed between the primary query and each vice modality (K, V), and the resulting features are fused with the self-attention outputs of the primary modality to enrich the representation capability. This process is repeated three times to generate cross-modal features Ft1’, Ft2’ and Ft2-fs' corresponding to T1, T2, and T2-FS, respectively. Each of these features is processed through an independent fully connected layer followed by a softmax function to obtain sequence-specific differentiation probabilities Pt1, Pt2 and Pt2-fs. Details of the implementation are provided in the Supplementary Figure S6. Key formulas used in SFM are shown below, using T1 as the primary modality example. $Q_{i}=w_{\mathrm{qi}}\cdot F_{i}$,$K_{i}=w_{\mathrm{ki}}\cdot F_{i}$,$V_{i}=w_{\mathrm{vi}}\cdot F_{i}$, I ∈ {t1’, t2’, t2-fs’}.

$F_{i}=MLP(softmax\left( \frac{Q_{t1’}k_{i}^{T}}{\sqrt{d_{k}}} \right)V_{i})$, i∈{t1’, t2’, t2-fs’}

$$Pi=MaxPool\left( \mathrm{add}\left( F_{t1’},F_{t2’},F_{t2-\mathrm{fs}’} \right) \right)$$

**
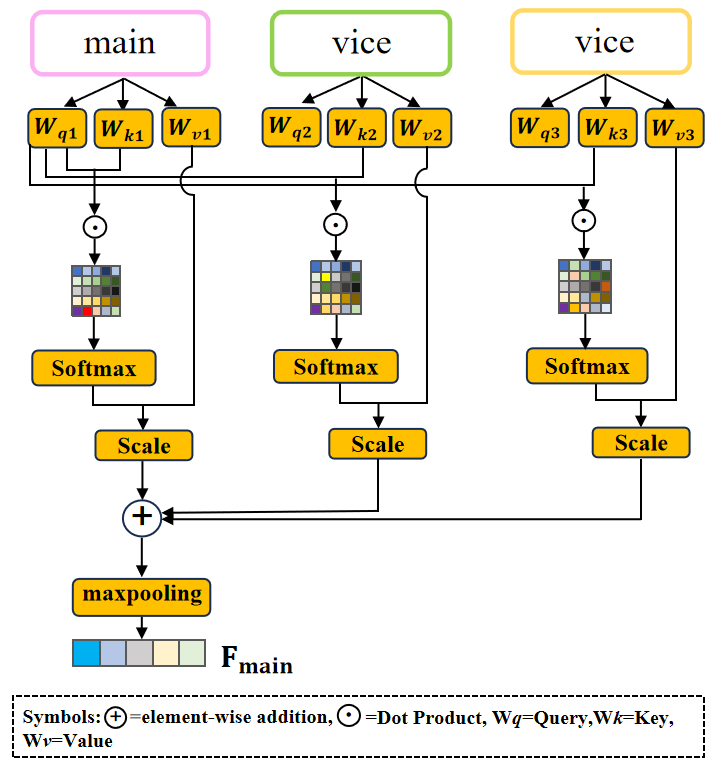
**

**Figure S4**. Overview of the proposed Sequence Fusion Module (SFM) architecture.

MIM

Figure S5 shows the architecture of the multi-plane fusion module (MIM), which is designed to integrate feature representations from different imaging planes (sagittal and axial) to capture cross-plane spatial dependencies and enhance spatial awareness. Initially, sagittal features Ft1’’, Ft2’’ and Ft2-fs’’ are weighted by a gating signal w and aggregated to form a fused sagittal representation Fsag. This representation is then combined with the axial feature Ftra through a MIM. Specifically, are first merged via element-wise addition to enhance semantic alignment. The fused feature is subsequently processed by two consecutive 1×1 convolutional layers with activation functions applied after each to refine channel-wise mappings and capture localized spatial cues. A Softmax operation is then applied to normalize the feature map, generating a probability distribution that guides classification decisions. Finally, an element-wise multiplication between the Softmax output and the preliminary fused features yields a plane-level prediction probability. This probability is then combined with the sagittal fusion result to construct a plane-aware abnormality matrix, enabling probability-level fusion. The key formulas used in the MIM are as follows:​

$$z_{put1}=softmax(Conv\left( \mathrm{Conv}\left( cat\left( Fsag,Faxial \right) \right) \right))$$

$$z_{put2}=MLP\left( add\left( Fsag,Faxial \right) \right))$$

$$Pp=MLP(softmax\left( z_{put1}\cdot z_{put2} \right))$$

**
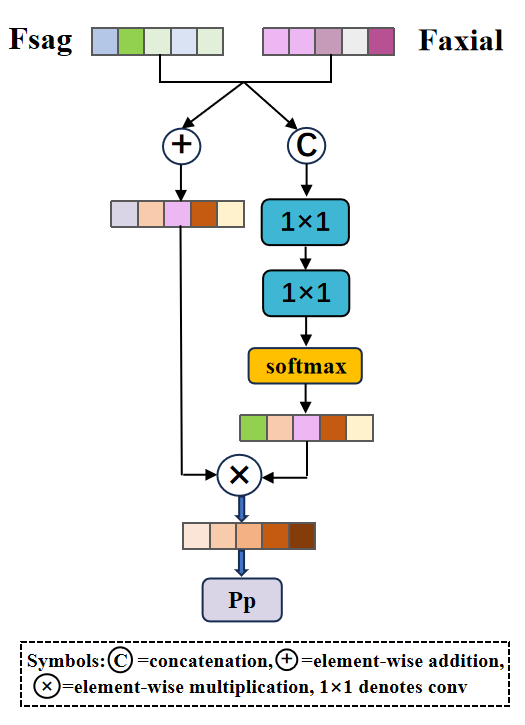
**

**Figure S5**. Structure of the plane-level fusion module (MIM), which adaptively combines sagittal and axial features to produce the final plane-aware representation.

SAMM and PAMM

Supplementary Fig. S6 illustrates the structure of the Sequence- (SAMM)^(5)^ and Plane-Aware Anomaly Matrix Module (PAMM). The SAMM captures complementary features among different MRI sequences (T1, T2 and T2-FS). Specifically, ISMF-Net calculates pairwise dot-product interactions between the predicted probabilities (pt1, pt2 and pt2-fs) derived from fused sequence features, generating a 5×5×5 anomaly probability matrix that quantifies inter-sequence relationships. Diagonal elements representing self-relations are extracted. The sequence-aware anomaly matrix is subsequently processed by a fully connected layer and a five-channel convolutional layer, and the final sequence-level probability Ps is obtained by weighting the matrix with the diagonal elements.

The PAMM captures spatial dependencies between different imaging planes (sagittal and axial). Pairwise dot-product operations on the predicted probabilities (Psag and Ptra) from fused multi-plane features generate a 5×5 anomaly probability matrix. Diagonal elements are excluded, and the matrix is refined by a fully connected layer and a four-channel convolutional layer. The final probability PI integrates sequence- and plane-level information for classification prediction. The key formulas used in the SAMM and PAMM are as follows:

$$Ps=Conv(Conv(MaxPool(z_{\mathrm{sag}})))\cdot z_{\mathrm{sagd}}$$

$$PI=Conv(Conv(Flatten(z_{\mathrm{tra}})))\cdot z_{\mathrm{trad}}$$

Here,$z_{\mathrm{sag}}\mathrm{and}z_{\mathrm{sagd}}$ represent the sequence anomaly matrix and its diagonal elements;$z_{\mathrm{tra}}\mathrm{and}z_{\mathrm{trad}}$ represent the plan anomaly matrix and its diagonal elements.


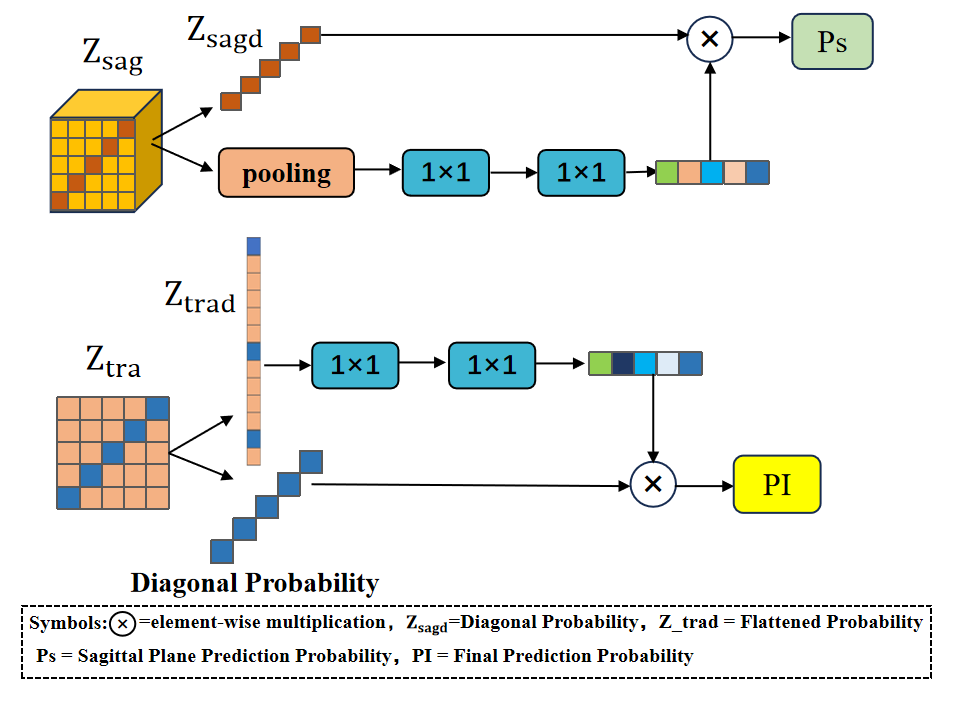


**Figure S6**. Illustration of the construction of sequence (SAMM) and plane-aware anomaly matrix modules (PAMM).

Performance Metrics

We adopted micro-accuracy as an overall performance metric, defined as the ratio of total correctly predicted samples across all classes to the total number of predictions:

$Micro-A\mathrm{ccuracy}=\frac{\sum_{c=1}^{C} \mathrm{TP}_{c}}{\sum_{c=1}^{C} (\mathrm{TP}_{c}+\mathrm{FP}_{c}+\mathrm{FN}_{c})}$​

Here, C is the total number of classes, and TPc, FPc and FNc refer to the true positives, false positives, and false negatives of class c, respectively.

Experimental

The total number of parameters of ISMF-Net is 201M, and a fixed, reproducible training and regularization protocol was adopted to mitigate the risk of overfitting under limited data conditions.

#### **Parameter initialization.**

Convolutional and linear layers were initialized with Kaiming initialization and zero biases, which matches ReLU activations by scaling the variance with fan-in to maintain stable signal propagation. Batch normalization parameters were initialized with scale γ = 1 and shift β = 0, making them equivalent to identity mappings at the beginning of training to avoid additional bias.

#### **Training protocol.**

ISMF-Net was optimized with AdamW using an initial learning rate of 3e-4. The decoupled weight decay in AdamW was set to 1e-4 and applied only to weight parameters (excluding biases and normalization parameters) to impose explicit regularization and suppress overfitting. The exponential decay rates for the first- and second-order moments were set to 0.9 and 0.99, respectively, to stabilize training. The batch size was 32, and training was performed for a maximum of 500 epochs. The learning rate schedule consisted of a 5-epoch linear warm-up (from 0 to 3e-4) followed by cosine annealing, gradually decaying the learning rate to a minimum of 1e-6.

#### **Regularization.**

Two explicit strategies were applied: (1) L2 weight decay through AdamW, and (2) dropout. In the backbone feature extraction module of ISMF-Net, dropout layers with p = 0.3 were inserted after activations within convolutional blocks. In the task head, a dropout layer with p = 0.5 was added before the final linear projection to reduce over-reliance on a small subset of discriminative channels.

#### **Early stopping.**

Training was terminated if the primary validation metric failed to improve by more than 1e-4 over 20 consecutive epochs, and the best-performing weights were restored.

#### **Data partitioning and evaluation.**

An external validation set was separated at the outset, while the remaining data were fixedly divided into training, validation, and testing sets. All experiments, including baseline comparisons, used exactly the same splits for training and evaluation. Data augmentation was applied exclusively to the training set to improve robustness and prevent information leakage into the validation and testing sets.

#### **Rationale.**

These measures are particularly suited for limited-data scenarios: AdamW’s decoupled weight decay provides explicit parameter constraints; the staged learning rate schedule (warm-up plus cosine annealing) reduces variance and oscillation; Kaiming initialization and unit-initialized batch normalization stabilize early gradient propagation; hierarchical dropout and early stopping together prevent overfitting to noise and spurious patterns; and data augmentation explicitly injects task invariances and increases effective sample diversity. Moreover, fixed dataset splits with an independent external validation set enable unbiased out-of-distribution evaluation and avoid optimistic bias.

Results

Patients

Table S5 illustrates the distribution of multi-sequence slice samples from each set for ISMF-Net. To prevent consecutive MRI scans from the same subject from being randomly assigned to both the training and validation sets, data splitting was still performed at the subject level, with subjects randomly divided into the training, validation, and test sets.

**Table S5. Distribution of multi-sequence slice samples from each set.**

|  | **Training set** | **Validation set** | **Internal test set** | **External test set** |
| --- | --- | --- | --- | --- |
| Total | 2716 (4170) | 447 | 869 | 981 |
| Schwannomas | 583 (875) | 128 | 248 | 274 |
| Meningiomas | 513 (855) | 60 | 120 | 251 |
| Astrocytoma | 387 (774) | 65 | 113 | 193 |
| Ependymomas | 433 (866) | 70 | 140 | 263 |
| Metastasis | 800 (800) | 124 | 248 | 406 |

Note: Each sample corresponds to four MR sequences (sagittal T1, sagittal T2, sagittal T2-FS, and axial T2) from one slice of a patient. The dataset was first split at the patient level in a 7:1:2 ratio, with minor deviations in slice-group counts due to variations in the number of slices per patient; data augmentation was then applied within each set to avoid data leakage.

Performance of the ISMF-Net

Figure S7 illustrates the ROC curves of ISMF-Net on the training set, internal test set, and external test set. The ROC curves for all three sets are above the chance level (AUC > 0.5), demonstrating the robustness and effectiveness of the proposed model.


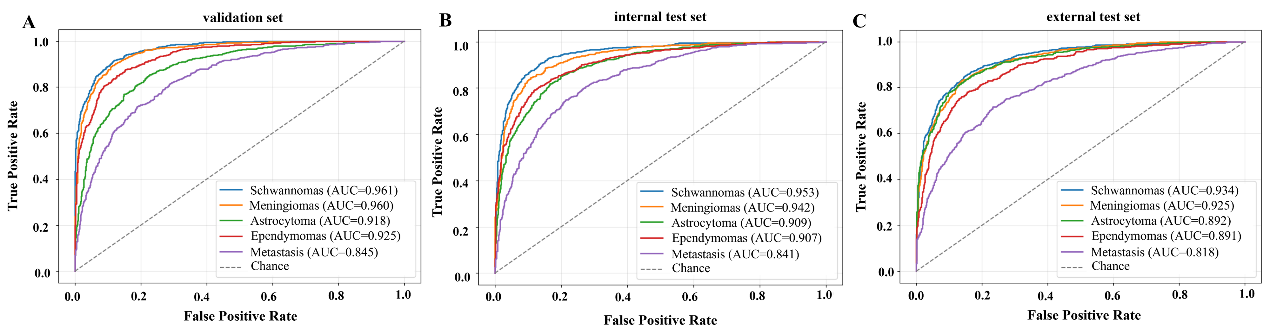


**Figure S7**. Receiver operating characteristic (ROC) curves of ISMF-Net for differentiating five types of intraspinal tumors (ISTs) on the validation (A), internal test (B), and external test (C) sets.

Figure S8 presents the confusion matrices of ISMF-Net on the (A) validation set, (B) internal test set, and (C) external test set. Each matrix illustrates the correspondence between the predicted and true labels across the five major types of intraspinal tumors. The results indicate that ISMF-Net maintains high consistency across all datasets. The major misclassifications occurred among AST, EPN, and metastases (MET), as well as between SCN and MNG. These misclassifications correspond to well-recognized diagnostic challenges in clinical practice, primarily arising from the substantial overlap in their imaging characteristics. Notably, these misclassification patterns were consistently observed across the validation, internal test, and external test cohorts, and were largely consistent with the diagnostic errors made by radiologists, suggesting that these errors mainly stem from the intrinsic diagnostic difficulty of these tumor entities rather than model-specific bias.


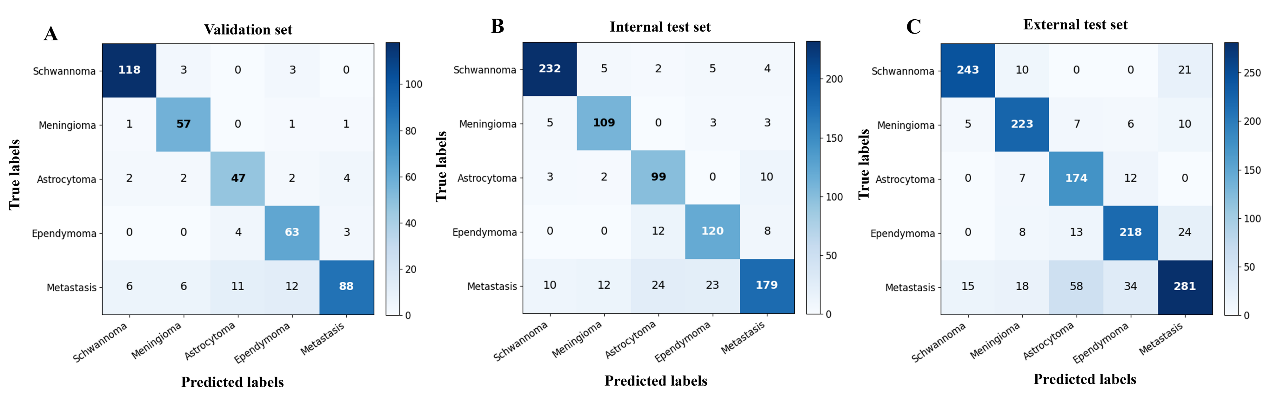


**Figure S8**. Confusion matrices of ISMF-Net on the validation (A), internal test (B), and external test (C) sets.

State-of-the-art methods

To ensure the fairness and reproducibility of the comparative experiments, the dataset was initially divided into a completely independent external validation set, while the remaining data were fixedly split into training, validation, and testing sets in a ratio of 7:1:2. Throughout the process, the external validation, validation, and testing sets were never involved in training or hyperparameter tuning, and once determined, the splits remained consistent across all experiments. Data augmentation was applied exclusively to the training set and in a uniform manner across all models, with the aim of enhancing robustness and preventing information leakage into the validation and testing sets. Both the proposed method and all comparison models were trained on identical training data under the same protocol, including the optimizer, learning rate schedule, batch size, number of epochs, regularization strategies, and early stopping criteria. Evaluation was performed on the same validation and testing sets using consistent metrics and calculation methods. The comparison models were implemented using either their official code or widely adopted open-source re-implementations, and all experiments were conducted within the same software and hardware environment. When recommended hyperparameters were provided in the official implementations or original publications, they were strictly followed; otherwise, default settings from public implementations were adopted.

Ablation study

We gradually remove modules and perform differentiation evaluation using simple concatenation and summation. We compare GLAFM with classical attention mechanisms and find that, for our ISTs problem, GLAFM shows better results in terms of overall metrics.

**Table S6. Performance comparison between the GLAFM and existing attention mechanisms.**

|  | **Precision** | **Recall** | **F1-score** | **micro-ACC** |
| --- | --- | --- | --- | --- |
| SE^(6)^ | 0.756[0.729,0.784] | 0.784[0.756,0.811] | 0.764[0.737,0.792] | 0.771[0.744,0.797] |
| ECA^(7)^ | 0.795[0.770,0.822] | 0.821[0.795,0.847] | 0.804[0.7780.830] | 0.809[0.784,0.834] |
| CBAM^(8)^ | 0.815[0.791,0.841] | 0.840[0.815,0.865] | 0.824[0.798,0.849] | 0.830[0.805,0.854] |
| GLAFM | 0.835[0.811,0.859] | 0.858[0.834,0.881] | 0.843[0.819,0.867] | 0.849[0.826,0.874] |

Note: Data are presented as mean [95% confidence interval]. Abbreviations: Micro-ACC, micro-averaged accuracy.

Observer study

Comparison of the radiologist group's average performance with and without ISMF-Net assistance.

Seven radiologists, including two seniors, two intermediates, and three juniors, participated in the observer study. Table S7 summarizes their overall diagnostic performance with and without model assistance. Model assistance consistently improved accuracy, sensitivity, and specificity across all readers, with the most notable gains in junior radiologists, moderate improvements in intermediates, and slight but stable enhancements in seniors. Table S8 presents the individual diagnostic performance of each radiologist under both conditions.

Figure S8 shows the Cohen’s Kappa heatmap illustrating the inter-rater agreement among seven radiologists as well as their agreement with the ground truth, before and after model assistance. Without model assistance, agreement levels varied substantially across radiologists, with relatively lower concordance observed between junior and senior radiologists, and weaker consistency with the ground truth. After model assistance, overall agreement improved markedly across all experience levels. Notably, the consistency between junior and intermediate radiologists, and between junior and senior radiologists, increased significantly, and the agreement with the ground truth also improved. These findings demonstrate that the proposed model enhances diagnostic consistency across radiologists of different experience levels while aligning more closely with the reference standard.

**Table S7. Comparison of the radiologist group's average performance with and without DL model assistance in the external test set.**

|  | **Accuracy** | | **Sensitivity** | | **Specificity** | |
| --- | --- | --- | --- | --- | --- | --- |
|  | **without** | **with** | **without** | **with** | **without** | **with** |
| **Radiologists average** | 0.882 [0.879, 0.886] | 0.925 [0.922, 0.928] | 0.719 [0.710, 0.728] | 0.827 [0.820, 0.834] | 0.926 [0.924, 0.929] | 0.953 [0.951, 0.955] |
| Schwannomas | 0.924 [0.919, 0.929] | 0.957 [0.953, 0.961] | 0.783 [0.764, 0.801] | 0.877 [0.862, 0.892] | 0.959 [0.955, 0.963] | 0.977 [0.973, 0.980] |
| Meningiomas | 0.907 [0.902, 0.914] | 0.950 [0.946, 0.954] | 0.811 [0.792, 0.829] | 0.881 [0.866, 0.896] | 0.929 [0.924, 0.935] | 0.965 [0.961, 0.969] |
| Astrocytoma | 0.872 [0.866, 0.878] | 0.917 [0.912, 0.922] | 0.693 [0.668, 0.717] | 0.846 [0.826, 0.865] | 0.901 [0.895, 0.907] | 0.928 [0.923, 0.934] |
| Ependymomas | 0.890 [0.884, 0.896] | 0.934 [0.929, 0.938] | 0.736 [0.716, 0.755] | 0.845 [0.828, 0.862] | 0.926 [0.920, 0.932] | 0.954 [0.949, 0.959] |
| Metastasis | 0.817 [0.810, 0.824] | 0.866 [0.860, 0.873] | 0.574 [0.556, 0.593] | 0.687 [0.670, 0.704] | 0.917 [0.911, 0.924] | 0.940 [0.935, 0.946] |

Note: The first-row average represents the macro-average across the following tumor types. For the remaining rows, each value represents the macro-averaged performance across the seven radiologists, with 95% confidence intervals provided for Accuracy, Sensitivity, and Specificity.

**Table S8. Comparison of the radiologist group's performance with and without DL model assistance in the external test set.**

|  | **Accuracy** | | **Sensitivity** | | **Specificity** | |
| --- | --- | --- | --- | --- | --- | --- |
|  | **without** | **with** | **without** | **with** | **without** | **with** |
| **Senior 1** | 0.926 [0.918,0.934] | 0.934 [0.926,0.942] | 0.829 [0.809,0.848] | 0.850 [0.832,0.869] | 0.954 [0.949,0.959] | 0.957 [0.954,0.963] |
| Schwannomas | 0.955 [0.943,0.965] | 0.962 [0.950,0.971] | 0.891 [0.848,0.921] | 0.898 [0.856,0.928] | 0.971 [0.960,0.980] | 0.978 [0.967,0.985] |
| Meningiomas | 0.950 [0.938,0.961] | 0.952 [0.939,0.962] | 0.888 [0.843,0.922] | 0.892 [0.848,0.925] | 0.964 [0.951,0.973] | 0.965 [0.952,0.974] |
| Astrocytoma | 0.918 [0.902,0.931] | 0.928 [0.913,0.940] | 0.829 [0.770,0.876] | 0.876 [0.822,0.915] | 0.932 [0.916,0.945] | 0.936 [0.921,0.949] |
| Ependymomas | 0.937 [0.923,0.949] | 0.951 [0.938,0.961] | 0.840 [0.791,0.880] | 0.878 [0.833,0.912] | 0.960 [0.947,0.970] | 0.968 [0.956,0.977] |
| Metastasis | 0.869 [0.850,0.886] | 0.876 [0.858,0.892] | 0.695 [0.648,0.737] | 0.707 [0.661,0.749] | 0.941 [0.924,0.954] | 0.946 [0.930,0.958] |
| **Senior 2** | 0.925 [0.917,0.933] | 0.928 [0.921,0.936] | 0.826 [0.807,0.845] | 0.836 [0.816,0.855] | 0.953 [0.948,0.958] | 0.955 [0.950,0.960] |
| Schwannomas | 0.957 [0.946,0.967] | 0.960 [0.949,0.969] | 0.887 [0.844,0.919] | 0.894 [0.852,0.925] | 0.975 [0.964,0.983] | 0.977 [0.966,0.984] |
| Meningiomas | 0.952 [0.940,0.962] | 0.955 [0.943,0.965] | 0.888 [0.843,0.922] | 0.896 [0.853,0.928] | 0.967 [0.954,0.976] | 0.968 [0.956,0.977] |
| Astrocytoma | 0.920 [0.905,0.933] | 0.924 [0.908,0.936] | 0.829 [0.770,0.876] | 0.845 [0.787,0.889] | 0.935 [0.919,0.947] | 0.936 [0.921,0.949] |
| Ependymomas | 0.932 [0.917,0.944] | 0.934 [0.919,0.946] | 0.837 [0.787,0.876] | 0.852 [0.804,0.890] | 0.954 [0.940,0.965] | 0.953 [0.939,0.964] |
| Metastasis | 0.862 [0.843,0.879] | 0.868 [0.849,0.885] | 0.690 [0.643,0.733] | 0.692 [0.646,0.736] | 0.934 [0.916,0.948] | 0.941 [0.924,0.954] |
| **Intermediate 1** | 0.899 [0.890,0.908] | 0.920 [0.912,0.929] | 0.760 [0.738,0.783] | 0.815 [0.796,0.835] | 0.936 [0.931,0.942] | 0.950 [0.945,0.955] |
| Schwannomas | 0.930 [0.915,0.942] | 0.954 [0.942,0.964] | 0.803 [0.752,0.846] | 0.880 [0.836,0.913] | 0.961 [0.948,0.971] | 0.972 [0.961,0.980] |
| Meningiomas | 0.928 [0.913,0.940] | 0.949 [0.936,0.959] | 0.817 [0.764,0.860] | 0.873 [0.826,0.908] | 0.952 [0.938,0.963] | 0.966 [0.953,0.975] |
| Astrocytoma | 0.897 [0.880,0.912] | 0.916 [0.900,0.929] | 0.777 [0.713,0.830] | 0.819 [0.758,0.867] | 0.916 [0.899,0.931] | 0.931 [0.916,0.944] |
| Ependymomas | 0.904 [0.887,0.919] | 0.927 [0.912,0.940] | 0.760 [0.705,0.808] | 0.829 [0.779,0.870] | 0.938 [0.922,0.950] | 0.950 [0.936,0.961] |
| Metastasis | 0.835 [0.814,0.854] | 0.857 [0.837,0.874] | 0.643 [0.595,0.688] | 0.677 [0.630,0.721] | 0.914 [0.895,0.930] | 0.931 [0.913,0.945] |
| **Intermediate 2** | 0.898 [0.889,0.907] | 0.925 [0.917,0.932] | 0.761 [0.740,0.783] | 0.826 [0.806,0.845] | 0.936 [0.930,0.941] | 0.953 [0.948,0.958] |
| Schwannomas | 0.932 [0.917,0.944] | 0.959 [0.947,0.968] | 0.799 [0.748,0.842] | 0.876 [0.832,0.910] | 0.965 [0.952,0.974] | 0.979 [0.969,0.986] |
| Meningiomas | 0.923 [0.908,0.936] | 0.951 [0.938,0.961] | 0.837 [0.786,0.877] | 0.880 [0.780,0.883] | 0.942 [0.927,0.954] | 0.968 [0.955,0.976] |
| Astrocytoma | 0.902 [0.885,0.917] | 0.917 [0.902,0.931] | 0.777 [0.713,0.830] | 0.838 [0.780,0.883] | 0.922 [0.906,0.936] | 0.929 [0.915,0.944] |
| Ependymomas | 0.911 [0.894,0.925] | 0.929 [0.914,0.941] | 0.783 [0.730,0.829] | 0.844 [0.795,0.883] | 0.940 [0.925,0.953] | 0.949 [0.934,0.960] |
| Metastasis | 0.822 [0.801,0.841] | 0.867 [0.848,0.884] | 0.611 [0.563,0.657] | 0.692 [0.646,0.735] | 0.909 [0.890,0.926] | 0.939 [0.922,0.952] |
| **Junior 1** | 0.845 [0.835,0.856] | 0.920 [0.912,0.929] | 0.627 [0.601,0.652] | 0.816 [0.796,0.836] | 0.904 [0.897,0.910] | 0.950 [0.945,0.956] |
| Schwannomas | 0.903 [0.886,0.917] | 0.957 [0.945,0.966] | 0.704 [0.648,0.755] | 0.861 [0.815,0.897] | 0.951 [0.937,0.963] | 0.980 [0.970,0.987] |
| Meningiomas | 0.874 [0.855,0.890] | 0.950 [0.938,0.961] | 0.765 [0.709,0.813] | 0.873 [0.826,0.908] | 0.898 [0.879,0.914] | 0.967 [0.955,0.976] |
| Astrocytoma | 0.827 [0.806,0.846] | 0.906 [0.889,0.920] | 0.549 [0.479,0.618] | 0.834 [0.775,0.880] | 0.872 [0.852,0.890] | 0.917 [0.900,0.931] |
| Ependymomas | 0.849 [0.830,0.867] | 0.929 [0.914,0.941] | 0.646 [0.587,0.702] | 0.837 [0.787,0.876] | 0.897 [0.878,0.913] | 0.950 [0.936,0.961] |
| Metastasis | 0.774 [0.752,0.796] | 0.861 [0.842,0.878] | 0.468 [0.420,0.517] | 0.677 [0.630,0.721] | 0.901 [0.881,0.918] | 0.937 [0.920,0.950] |
| **Junior 2** | 0.845 [0.835,0.854] | 0.923 [0.915,0.931] | 0.624 [0.599,0.650] | 0.822 [0.803,0.842] | 0.903 [0.897,0.910] | 0.952 [0.947,0.957] |
| Schwannomas | 0.901 [0.884,0.915] | 0.955 [0.943,0.965] | 0.693 [0.636,0.745] | 0.872 [0.828,0.907] | 0.951[0.935, 0.963] | 0.976 [0.965,0.983] |
| Meningiomas | 0.869 [0.850,0.886] | 0.942 [0.929,0.953] | 0.765 [0.709,0.813] | 0.869 [0.821,0.905] | 0.892 [0.872,0.908] | 0.959 [0.945,0.969] |
| Astrocytoma | 0.827 [0.806,0.846] | 0.916 [0.901,0.930] | 0.549 [0.479,0.618] | 0.839 [0.781,0.884] | 0.872 [0.852,0.890] | 0.929 [0.913,0.942] |
| Ependymomas | 0.853 [0.833,0.871] | 0.932 [0.917,0.944] | 0.646 [0.587,0.702] | 0.840 [0.791,0.880] | 0.901 [0.882,0.917] | 0.953 [0.939,0.964] |
| Metastasis | 0.774 [0.751,0.795] | 0.870 [0.851,0.886] | 0.468 [0.420,0.517] | 0.690 [0.643,0.733] | 0.900 [0.880,0.917] | 0.944 [0.928,0.957] |
| **Junior 3** | 0.838 [0.828,0.848] | 0.922 [0.914,0.931] | 0.609 [0.583,0.634] | 0.824 [0.805,0.844] | 0.900 [0.894,0.906] | 0.952 [0.947,0.957] |
| Schwannomas | 0.892 [0.875,0.908] | 0.952 [0.940,0.962] | 0.703 [0.647,0.754] | 0.858 [0.811,0.894] | 0.939 [0.923,0.952] | 0.976 [0.965,0.983] |
| Meningiomas | 0.859 [0.839,0.876] | 0.949 [0.937,0.960] | 0.717 [0.658,0.769] | 0.883 [0.837,0.917] | 0.890 [0.870,0.907] | 0.964 [0.951,0.973] |
| Astrocytoma | 0.813 [0.792,0.833] | 0.911 [0.895,0.925] | 0.539 [0.468,0.608] | 0.870 [0.816,0.911] | 0.858 [0.837,0.876] | 0.918 [0.901,0.932] |
| Ependymomas | 0.844 [0.824,0.862] | 0.934 [0.919,0.946] | 0.639 [0.579,0.694] | 0.837 [0.787,0.876] | 0.892 [0.873,0.909] | 0.956 [0.943,0.967] |
| Metastasis | 0.782 [0.760,0.803] | 0.865 [0.846,0.882] | 0.446 [0.398,0.494] | 0.673 [0.626,0.717] | 0.921 [0.903,0.937] | 0.945 [0.929,0.957] |

Note: The first row for each radiologist represents the macro-average of the following tumor types. For the remaining rows, we provide the 95% confidence intervals for Accuracy, Sensitivity, and Specificity. Two senior radiologists (Senior 1: 30 and Senior 2: 20 years of experience), two intermediate radiologists (intermediate1:12 and intermediate 2: 11 years of experience), and three junior radiologists (junior 1:6, junior 2:4, and junior 3:1 year of experience, respectively).


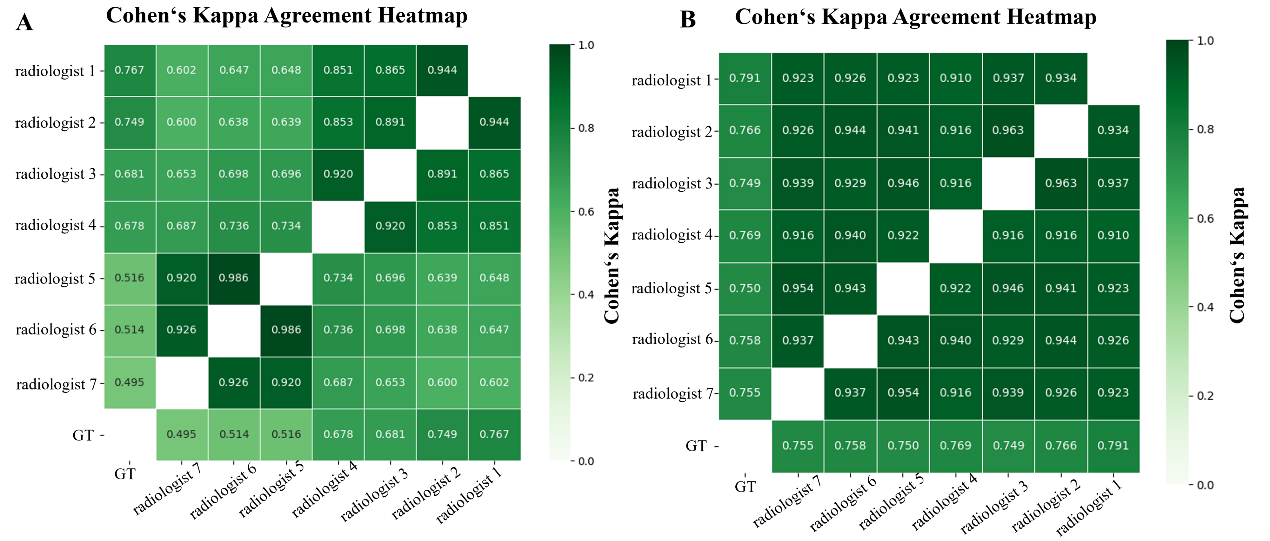


**Figure S9**. Cohen’s kappa heatmaps showing agreements among radiologists and the ground truth: without (A) and with (B) model assistance.

Analysis of sequence preference between radiologists and ISMF-Net.

We collected sequence preference data from senior radiologists based on clinical experience (Table S9), and compared it with ISMF-Net’s single-sequence F1-scores obtained by independently evaluating the predictions from the T1, T2, and T2-FS branches. Figure S10, S11 presents the correlation analysis between ISMF-Net’s performance and clinical preferences for each ISTs. The results demonstrate a high degree of consistency between ISMF-Net’s single-sequence evaluation and expert clinical judgment, supporting the rationality and scientific validity of our approach.

**Table S9. Summary of sequence preference comparison between radiologists and ISMF-Net.**

|  | **T1W** | **T2W** | **T2-FS** |
| --- | --- | --- | --- |
| **Radiologists** |  |  |  |
| Schwannoma | 0.500 | 0.900 | 0.850 |
| Meningioma | 0.650 | 0.750 | 0.400 |
| Astrocytoma | 0.600 | 0.950 | 0.800 |
| Ependymoma | 0.750 | 0.900 | 0.650 |
| Metastasis | 0.850 | 0.750 | 0.950 |
| **ISMF-Net** |  |  |  |
| Schwannoma | 0.635 | 0.850 | 0.800 |
| Meningioma | 0.680 | 0.812 | 0.600 |
| Astrocytoma | 0.505 | 0.683 | 0.655 |
| Ependymoma | 0.701 | 0.750 | 0.683 |
| Metastasis | 0.600 | 0.621 | 0.660 |

Note: T1, T1-weighted imaging; T2, T2-weighted imaging; T2-fs, T2 fat-suppressed imaging.


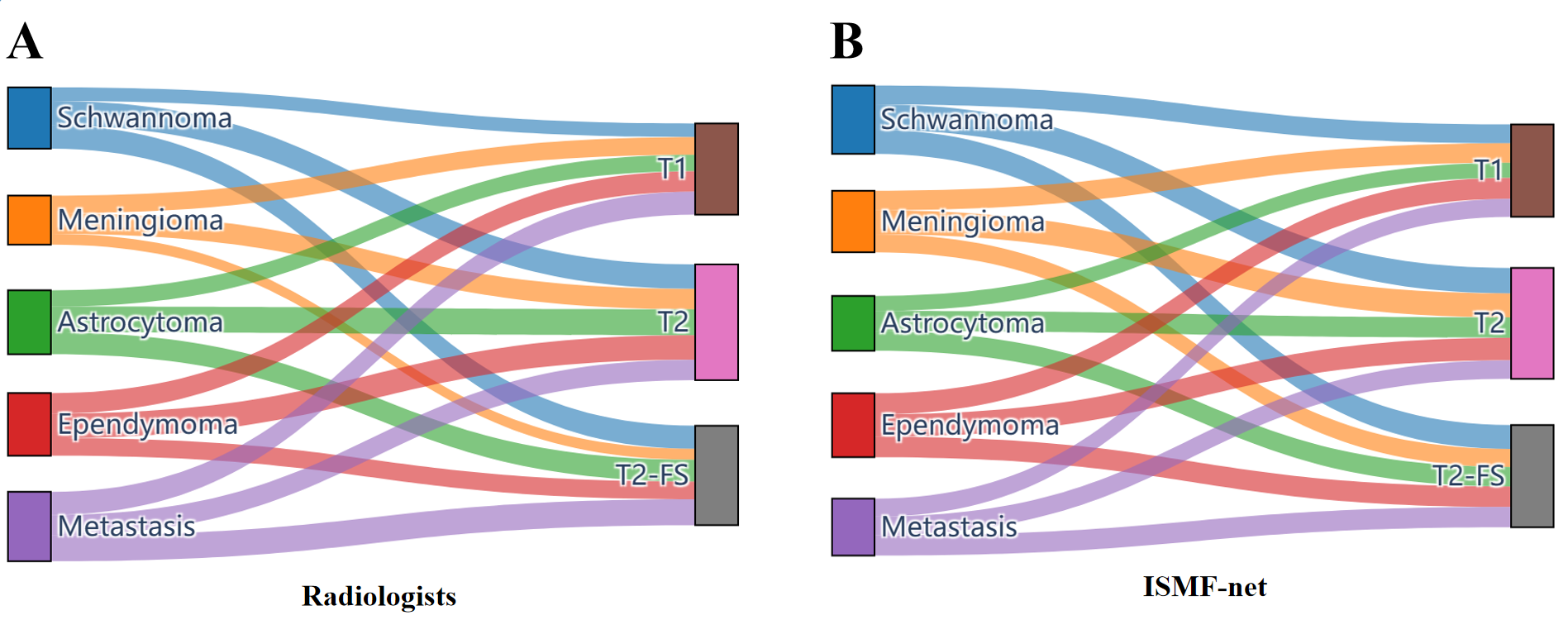


**Figure S10**. Sequence preference analysis of different intraspinal tumors (ISTs). (A) Sankey diagram illustrating the distribution of radiologists’ preferred MRI sequences (T1, T2, and T2-FS) for diagnosing five ISTs. (B) Model performance (F1-score) across different MRI sequences. Different colors represent distinct ISTs, with line thickness indicating the likelihood of observing a particular abnormality within that specific sequence.


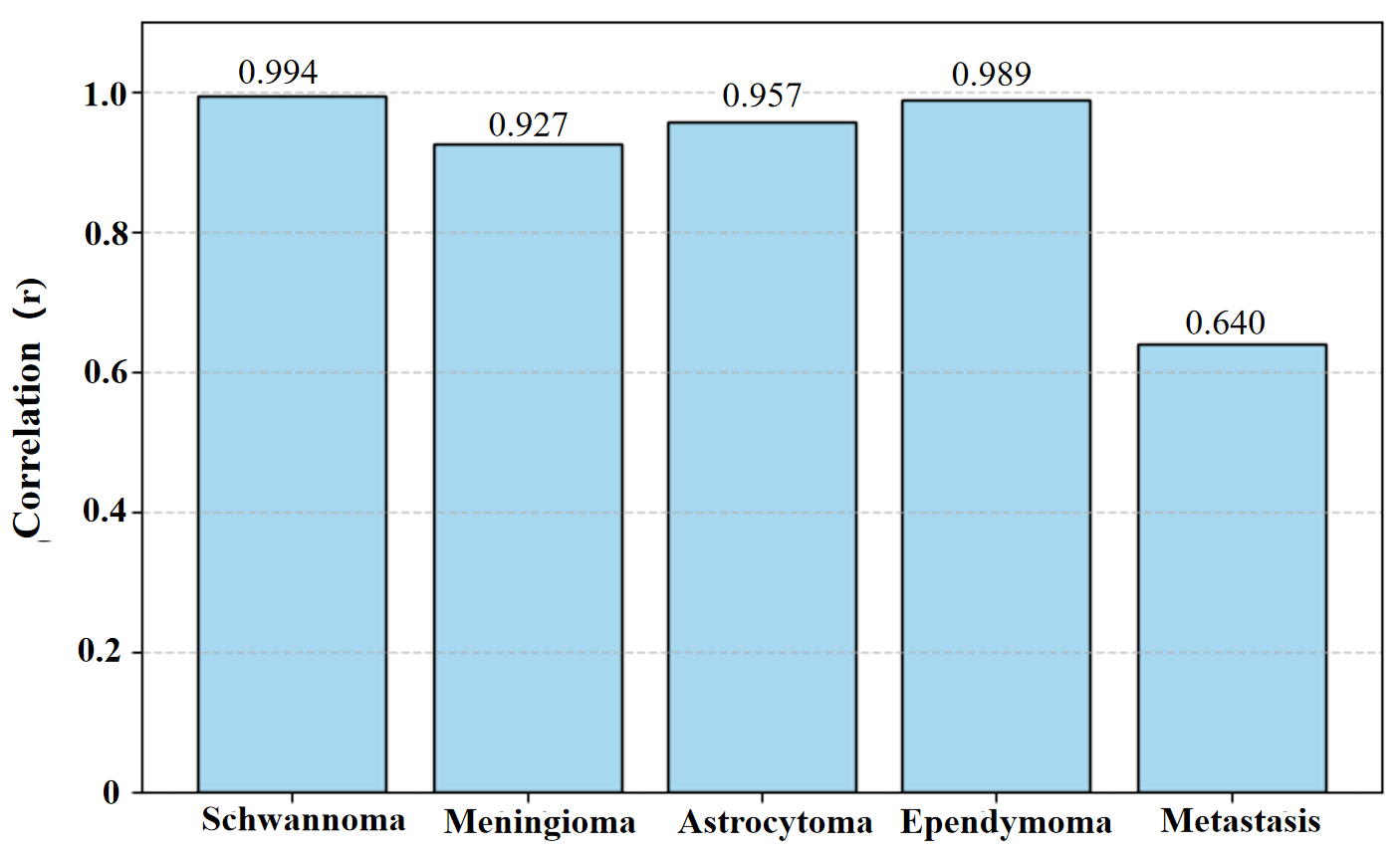


**Figure S11**. Correlation between model-predicted F1-scores and radiologists’ diagnostic preferences.

Plan preference analysis

Table S10 presents the spatial preference distribution and clinical sequence preference reported by radiologists. As shown, the relative contributions of each imaging plane are similar across different tumor subtypes.

**Table S10. Summary of plan preference comparison between radiologists and ISMF-Net.**

| **Radiologists** | **Sagittal** | **Axial** |
| --- | --- | --- |
| Schwannoma | 0.750 | 0.800 |
| Meningioma | 0.850 | 0.850 |
| Astrocytoma | 0.850 | 0.800 |
| Ependymoma | 0.850 | 0.800 |
| Metastasis | 0.780 | 0.800 |
| **ISMF-Net** |  |  |
| Schwannoma | 0.680 | 0.705 |
| Meningioma | 0.721 | 0.720 |
| Astrocytoma | 0.620 | 0.596 |
| Ependymoma | 0.752 | 0.750 |
| Metastasis | 0.600 | 0.632 |

Comparative case analysis of seven radiologists and ISMF-Net

Figure S12 illustrates the diagnostic results of the ISMF-net and radiologists for eight cases. ISMF-Net achieved accurate diagnoses in 1-5 cases, demonstrating consistent performance. In contrast, the radiologists' diagnostic results exhibited significant variability, with diagnostic accuracy positively correlated with experience level. The diagnoses of senior radiologists were generally in agreement with the ground truth. In Patient 3, Radiologist 2 misclassified an AST as MET, likely due to similarities in distribution and margin morphology between the two lesions. The diagnostic performance of intermediate radiologists fell between that of junior and senior radiologists, but misdiagnosis rates remained high for difficult lesions like METs. Junior radiologists nearly failed to provide correct diagnoses for all eight difficult cases, with only Radiologist 5 (6 years of experience) successfully diagnosing Patient 4. Notably, ISMF-Net demonstrated exceptional accuracy in detecting MET, effectively addressing challenges such as blurred boundaries and variable morphologies, with performance comparable to senior radiologists in these cases.

Patients 6 - 8 represent challenging cases of atypical AST and MET. Both ISMF-Net and junior radiologists failed to correctly identify the lesions, likely due to the high similarity between the lesions and surrounding normal tissue, as well as atypical signal characteristics. In addition, the inherent heterogeneity of MET can cause them to closely resemble other ISTs. In contrast, senior radiologists made accurate diagnoses in all three cases, demonstrating their superior stability and reliability when faced with difficult scenarios. These findings highlight ISMF-Net’s limited sensitivity to subtle distinctions between lesions and adjacent structures, underscoring the need for further optimization to improve its performance in diagnostically complex cases.


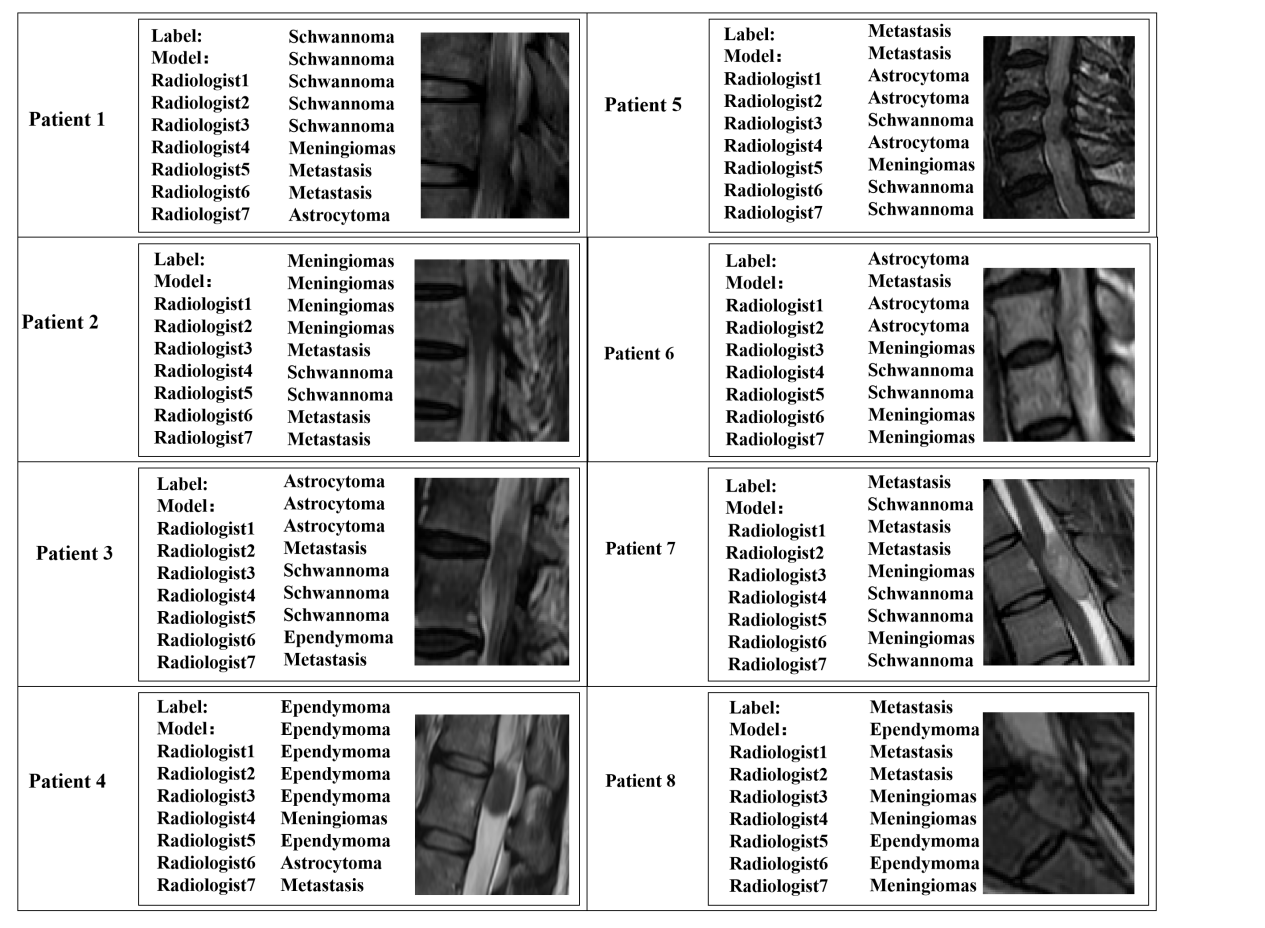


**Figure S12**. Representative diagnostic results for eight intraspinal tumor (IST) cases, comparing predictions from the proposed ISMF-Net model with ground truth labels and seven radiologists’ assessments.

References

1. Wang C-Y, Yeh I-H, Mark Liao H-Y. Yolov9: Learning what you want to learn using programmable gradient information. European conference on computer vision; 2024.

2. Qi Y, He Y, Qi X, Zhang Y, Yang G. Dynamic snake convolution based on topological geometric constraints for tubular structure segmentation. Proceedings of the IEEE/CVF international conference on computer vision; 2023 :6070-9.

3. Dai Y, Gao Y, Liu F. Transmed: Transformers advance multi-modal medical image classification. Diagnostics. 2021;11(8):1384.

4. Dai J, Qi H, Xiong Y, et al. Deformable convolutional networks. Proceedings of the IEEE international conference on computer vision, 2017:764-73.

5. Qiu Z, Xie Z, Lin H, et al. Learning co-plane attention across MRI sequences for diagnosing twelve types of knee abnormalities. Nature Communications. 2024;15(1):7637.

6. Hu J, Shen L, Sun G. Squeeze-and-excitation networks. Proceedings of the IEEE conference on computer vision and pattern recognition, 2018:7132-41.

7. Liu T, Luo R, Xu L, et al. Spatial channel attention for deep convolutional neural networks. Mathematics. 2022;10(10):1750.

8. Woo S, Park J, Lee J-Y, Kweon IS. Cbam: Convolutional block attention module. Proceedings of the European conference on computer vision (ECCV), 2018:3-19.
